# Genome-based analysis for the identification of genes involved in *o*-xylene degradation in *Rhodococcus opacus* R7

**DOI:** 10.1186/s12864-018-4965-6

**Published:** 2018-08-06

**Authors:** Alessandra Di Canito, Jessica Zampolli, Alessandro Orro, Pasqualina D’Ursi, Luciano Milanesi, Guido Sello, Alexander Steinbüchel, Patrizia Di Gennaro

**Affiliations:** 1 Department of Biotechnology and Biosciences, University of Milano-Bicocca, Piazza della Scienza 2, 20126 Milan, Italy; 2 ITB, CNR, via Fratelli Cervi 19, 20133 Segrate, Milan, Italy; 3 Department of Chemistry, University of Milano, via Golgi 19, 20133 Milan, Italy; 4 Department of Molecular Microbiology and Biotechnology, Westfälische Wilhelms-Universität Münster, Münster, Germany; 5 Environmental Sciences Department, King Abdulaziz University, Jeddah, Saudi Arabia

**Keywords:** *Rhodococcus*, Microbial genomics, *o*-xylene degradation, *Gene clusters*, Contaminated soil

## Abstract

**Background:**

Bacteria belonging to the *Rhodococcus* genus play an important role in the degradation of many contaminants, including methylbenzenes. These bacteria, widely distributed in the environment, are known to be a powerhouse of numerous degradation functions, due to their ability to metabolize a wide range of organic molecules including aliphatic, aromatic, polycyclic aromatic compounds (PAHs), phenols, and nitriles. In accordance with their immense catabolic diversity, *Rhodococcus* spp. possess large and complex genomes, which contain a multiplicity of catabolic genes, a high genetic redundancy of biosynthetic pathways and a sophisticated regulatory network. The present study aimed to identify genes involved in the *o*-xylene degradation in *R. opacus* strain R7 through a genome-based approach.

**Results:**

Using genome-based analysis we identified all the sequences in the R7 genome annotated as dioxygenases or monooxygenases/hydroxylases and clustered them into two different trees. The *akb*, *phe* and *prm* sequences were selected as genes encoding respectively for dioxygenases, phenol hydroxylases and monooxygenases and their putative involvement in *o*-xylene oxidation was evaluated. The involvement of the *akb* genes in *o*-xylene oxidation was demonstrated by RT-PCR/qPCR experiments after growth on *o*-xylene and by the selection of the R7–50 leaky mutant. Although the *akb* genes are specifically activated for *o*-xylene degradation, metabolic intermediates of the pathway suggested potential alternative oxidation steps, possibly through monooxygenation. This led us to further investigate the role of the *prm* and the *phe* genes. Results showed that these genes were transcribed in a constitutive manner, and that the activity of the Prm monooxygenase was able to transform *o*-xylene slowly in intermediates as 3,4-dimethylphenol and 2-methylbenzylalcohol. Moreover, the expression level of *phe* genes, homologous to the *phe* genes of *Rhodococcus* spp. 1CP and UPV-1 with a 90% identity, could explain their role in the further oxidation of *o*-xylene and R7 growth on dimethylphenols.

**Conclusions:**

These results suggest that R7 strain is able to degrade *o*-xylene by the Akb dioxygenase system leading to the production of the corresponding dihydrodiol. Likewise, the redundancy of sequences encoding for several monooxygenases/phenol hydroxylases, supports the involvement of other oxygenases converging in the *o*-xylene degradation pathway in R7 strain.

**Electronic supplementary material:**

The online version of this article (10.1186/s12864-018-4965-6) contains supplementary material, which is available to authorized users.

## Background

Methylbenzenes are pollutants of great relevance for their toxic properties and their wide spread in environment, commonly present in crude petroleum and in various industrial processes [1]. Different methylbenzenes, including the three xylene isomers, can be degraded by several bacterial strains, with a degradation pathway that depends on the position of the methyl groups on the aromatic ring [2]. These bacteria can be divided into two groups: i) microorganisms that can degrade both *m*- and *p*-xylene; and ii) microorganisms that can only degrade the *ortho* isomer. The two degradation pathways are rarely found simultaneously in the same microorganism.

**Fig. 1:**
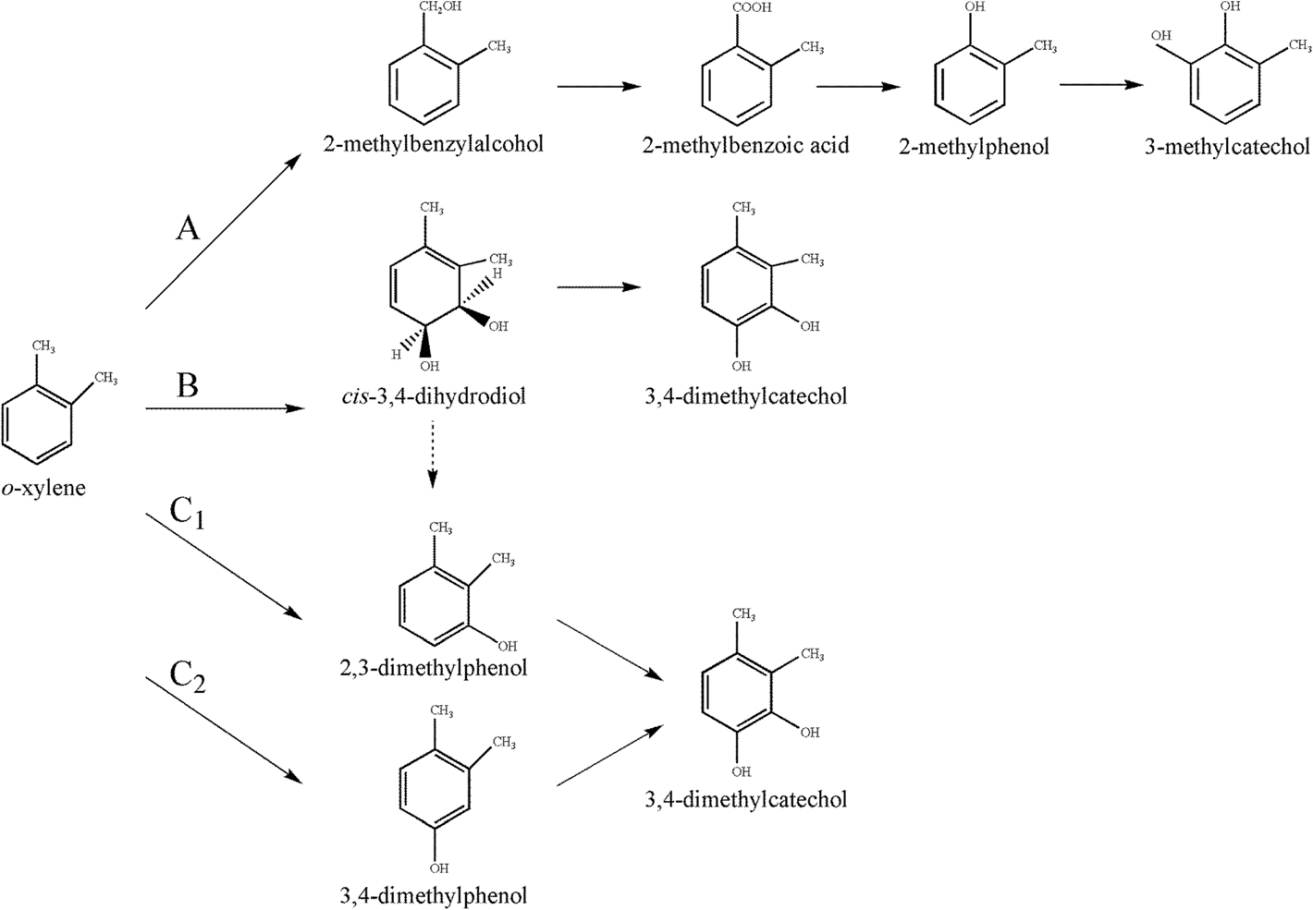
Overview of different *o*-xylene degradation pathways in bacteria. Monooxygenation of the methyl group and subsequent oxidations in *Rhodococcus* sp. strain B3 (route A); dioxygenation and subsequent dehydrogenation of the aromatic ring in *Rhodococcus* sp. strain DK17 (route B); two successive monooxygenation reactions of the aromatic ring in *P. stutzeri* strain OX1 (route C1 and C2). Dashed arrow indicates a spontaneous dehydration

Although bacteria capable of degrading *m*-xylene or *p*-xylene under aerobic conditions and their corresponding catabolic pathways have been well documented [3–5], bacteria capable of degrading *o*-xylene are not so common and their metabolic pathways have been poorly investigated [6–10]. Three *o*-xylene degradation pathways (leading to their corresponding catechols) have been reported [1, 8, 11–13] (Fig. 1). The first pathway is initiated by oxidation of the methyl group of *o*-xylene to form 2-methylbenzylalcohol, subsequently metabolized to form 3-methylcatechol as reported in the case of *Rhodococcus* sp. strain B3 [8] (Fig. 1, route A). The second degradation pathway of *o*-xylene is initiated by a ring-hydroxylating 2,3-dioxygenase leading to the 3,4-dimethylcatechol in *Rhodococcus* sp. strain DK17 (Fig. 1, route B) [10]. While the third pathway is initiated by a ring-hydroxylating monooxygenase at the different position 3, or 4, as performed by the Toluene *o*-xylene Monooxygenase (ToMo) in *P. stutzeri* strain OX1 (Fig. 1, routes C1 and C2), leading to the formation of 3,4-dimethylcatechol [1].

One of the main roles in the degradation of many contaminants, including methylbenzenes, is played by bacteria belonging to the *Rhodococcus* genus. These bacteria, widely distributed in the environment, are characterized as a powerhouse of numerous degradation functions, since they are able to metabolize a wide range of organic molecules including aliphatic, aromatic and polycyclic aromatic compounds (PAHs), phenols, and nitriles [14]. In accordance with their immense catabolic diversity, *Rhodococcus* spp. are characterized to possess large and complex genomes, which contain a multiplicity of catabolic genes, a high gene redundancy of biosynthetic pathways and a sophisticated regulatory network [15]. Many of them also possess a variety of large linear plasmids and smaller circular plasmids that contribute to and also explain the immense repertoire of catabolic abilities [16]. The most known example is represented by the genome of *Rhodococcus jostii* strain RHA1 [17], isolated for its ability to aerobically degrade polychlorinated biphenyls (PCBs) [18], and also able to utilize a wide range of compounds as sole carbon and energy source. Analyses of the 9.7 Mb large genome of RHA1 provided the evidence of catabolic pathway redundancy and horizontal gene transfer events [17].

To date, several gene clusters involved in the degradation of multiple aromatic compounds have been identified from genome analysis of several *Rhodococcus* spp. strains, including genes for biphenyl [19], isopropylbenzene and ethylbenzene [20] and methylbenzenes [2]. However, few not in depth genetic studies have been reported regarding the abilities of *Rhodococcus* strains to degrade *o*-xylene. The only data regarding the genes involved in *o*-xylene degradation in bacteria belonging to *Rhodococcus* genus derives from the identification of the *akb* genes in *Rhodococcus* sp. strain DK17 [10]. This strain is able to grow on *o*-xylene (and toluene, ethylbenzene, isopropylbenzene) through a multicomponent *o*-xylene dioxygenase [10, 12]. The DK17 *o*-xylene dioxygenase is described to perform a ring-oxidizing pathway leading to the 3,4-dimethylcathechol formation either by a dioxygenation or two monooxygenations, which can introduce two oxygen atoms successively. Thus, a deeper analysis concerning *o*-xylene degradation in *Rhodococcus* is necessary to understand which genes and enzymes could be involved in this metabolism.

In this context, the metabolically versatile *Rhodococcus opacus* R7 [21], known for its ability to grow on naphthalene, several long- and medium-chain *n*-alkanes, and aromatic hydrocarbons belonging to BTEX group (benzene, toluene, ethylbenzene and xylenes) [22, 23], and also able to grow on *o*-xylene, can be used to add information of the metabolism of this compound. The whole genome of R7 strain was completely sequenced and it revealed to possess multiple genes for the degradation of a large set of aliphatic, aromatic and PAHs compounds [24]. Moreover, the genome analysis revealed the presence, beside the chromosome, of five plasmids (pPDG1, pPDG2, pPDG3, pPDG4, pPDG5) that provided the evidence of high catabolic pathway redundancy.

Through a genome-based analysis, the present work aimed to identify genes and molecular mechanisms involved in *o*-xylene degradation in *R. opacus* R7. Based on the previous identification of 2,3-dimethylphenol (2,3-DMP) and 3,4-dimethylphenol (3,4-DMP) in the R7 culture medium and the fact that these intermediates were metabolized by R7 strain, when supplied as the sole carbon and energy source, we identified these compounds as intermediates of R7 *o*-xylene degradation pathway [21, 22]. However, literature data suggested that the formation of dimethylphenol could be attributed to the dehydration of dihydrodiol deriving from the dioxygenation activity when *o*-xylene is supplied to *Rhodococcus* sp. strain DK17 [2]. For this reason, we searched and identified in the R7 genome all the sequences annotated as dioxygenases or monooxygenases/hydroxylases and clustered them into two different trees in order to select the oxygenases putatively involved in the *o*-xylene oxidation. Moreover, we demonstrated that the selected genes were involved in *o*-xylene degradation in *R. opacus* R7 by RT-PCR/qPCR, mutant analysis, cloning and expression experiments, thus revealing the complexity of this metabolic network.

## Results

### R7 genome sequence analysis

The formation of 2,3-dimethylphenol and 3,4-dimethylphenol (and the oxidation to 2-methylbenzylalcohol) in the R7 *o*-xylene degradation pathway [21, 22] suggests the involvement of monooxygenases able to oxidize *o*-xylene. However, the only *o*-xylene degradation pathway described in literature for bacteria of *Rhodococcus* genus is through the dioxygenase system of *Rhodococcus* sp. DK17 leading to the corresponding dihydrodiol that could be dehydrated to DMPs [2]. Therefore, we hypothesized that in the case of *R. opacus* strain R7 the formation of the identified intermediates could be explained by the involvement of different oxygenase systems for *o*-xylene degradation. For this reason, first we identified all the sequences annotated as dioxygenases or monooxygenases/hydroxylases in the R7 genome and clustered them into two different trees in order to select all the oxygenases putatively involved in *o*-xylene oxidation.

#### Analysis and clusterization of dioxygenases

**Fig. 2:**
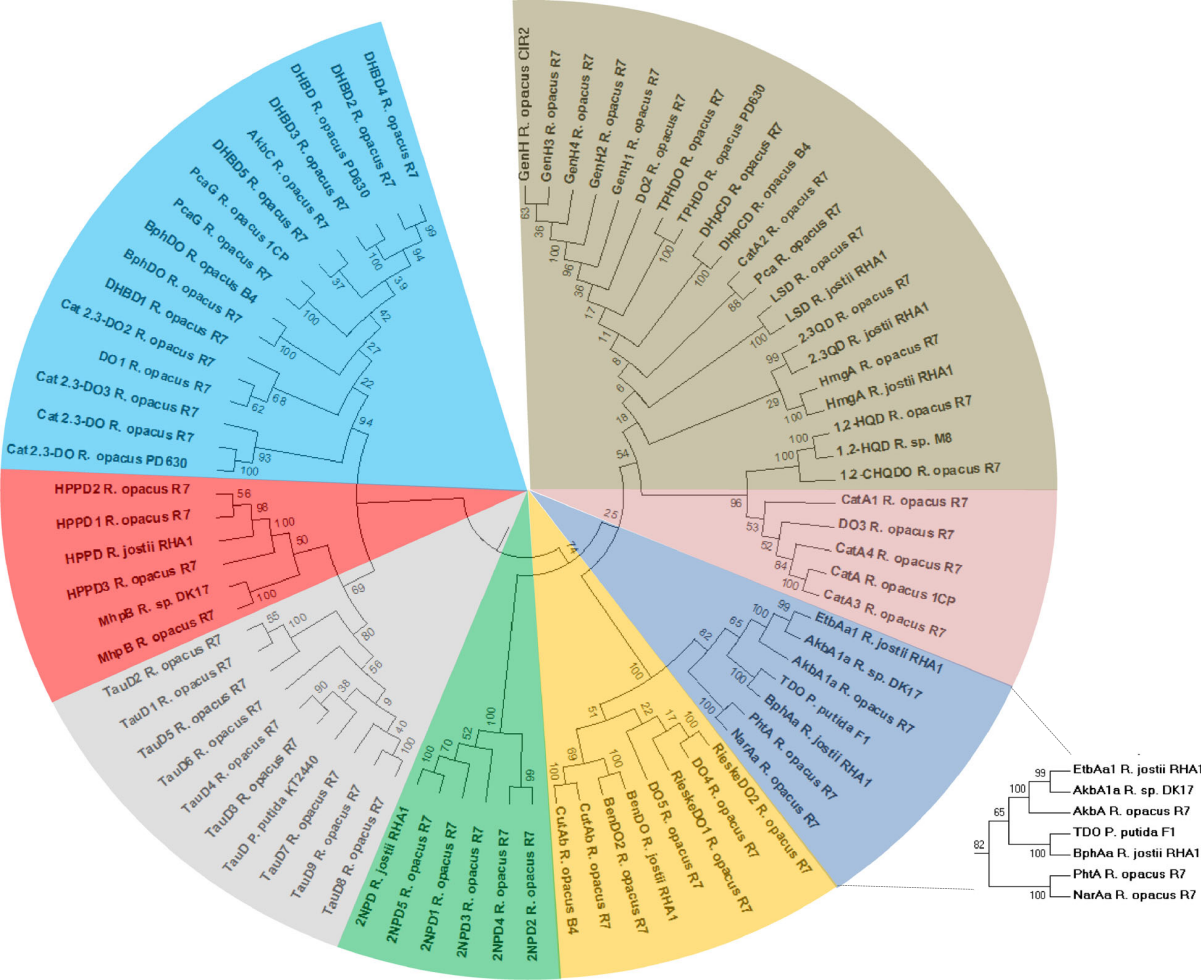
Phylogenetic tree of *R. opacus* R7 proteins containing the catalytic subunit of dioxygenases. The tree was constructed aligning selected reference protein sequences with R7 sequences. Numbers represent the bootstrap values on the branches calculated for maximum likelihood (ML) method selected from the package MEGA version 6 with 100 bootstraps. Color scheme for dioxygenases: brown, clade 1; pink, clade 2; blue, clade 3; yellow, clade 4; green, clade 5; grey, clade 6; red, clade 7; light blue, clade 8. The extended clade includes the AkbA1a of *R. opacus* R7 homologous to the AkbA1a of *R*. sp. DK17 involved in the oxidation of *o*-xylene. Abbreviation of dioxygenase names are reported in Additional file 1: Table S1

A preliminary genome RAST annotation of R7 allowed the identification of 83 potential dioxygenases. Among those, only 57 were selected as catalytic subunits of R7 dioxygenases. In order to cluster the R7 catalytic subunits, 22 reference sequences putatively involved in the aromatic compound degradation were considered. The generated tree reveals that these amino acid sequences are divided into eight clades (Fig. 2). All the sequences are listed in Additional file 1: Table S1, including the reference sequences with the relative source strain and all the sequences belonging to R7 strain.

Clade number 3 includes dioxygenases putatively involved in the upper pathways of BTEX compounds and polycyclic aromatic hydrocarbons (Fig. 2, blue box), while the other seven clades include all the dioxygenases putatively involved in the peripheral pathways of different aromatic compounds (Fig. 2).

Among the sequences in clade number 3 of the dioxygenase-tree (Fig. 2, extended clade), the catalytic subunit of ethylbenzene dioxygenase (EtbAa1) of *R*. *jostii* RHA1 [25] and the *o*-xylene dioxygenase (AkbA1a) of *Rhodococcus* sp. DK17 [10], are shown to cluster near the only homologous dioxygenase sequence (AkbA1a) of R7. Multiple alignments of AkbA1a of R7 with proteins belonging to Bacterial Rieske non-heme iron oxygenases reveals the coordination of the center iron-sulfur (Fe-S) (CxH - CxxH) with the amino acids that coordinate the iron atom of the active site (H - H - D). Moreover, the *akbA1a* gene encoding for the AkbA1a dioxygenase shows a nucleotide identity around 90% with the *etbAa1* of *R. jostii* RHA1 and *akbA1a* of *Rhodococcus* sp. DK17. For these features, the AkbA1a was taken into consideration for its involvement in *o*-xylene catabolism.

The remaining clades of the tree analysis include several dioxygenases involved in the mechanism of aromatic ring cleavage (e. g. gentisate 1,2-dioxygenases, catechol 1,2-dioxygenases, catechol 2,3-dioxygenases, homogentisate dioxygenase and protocatechuate dioxygenase), that are not taken into consideration.

#### Analysis and clusterization of monooxygenases/hydroxylases

**Fig. 3:**
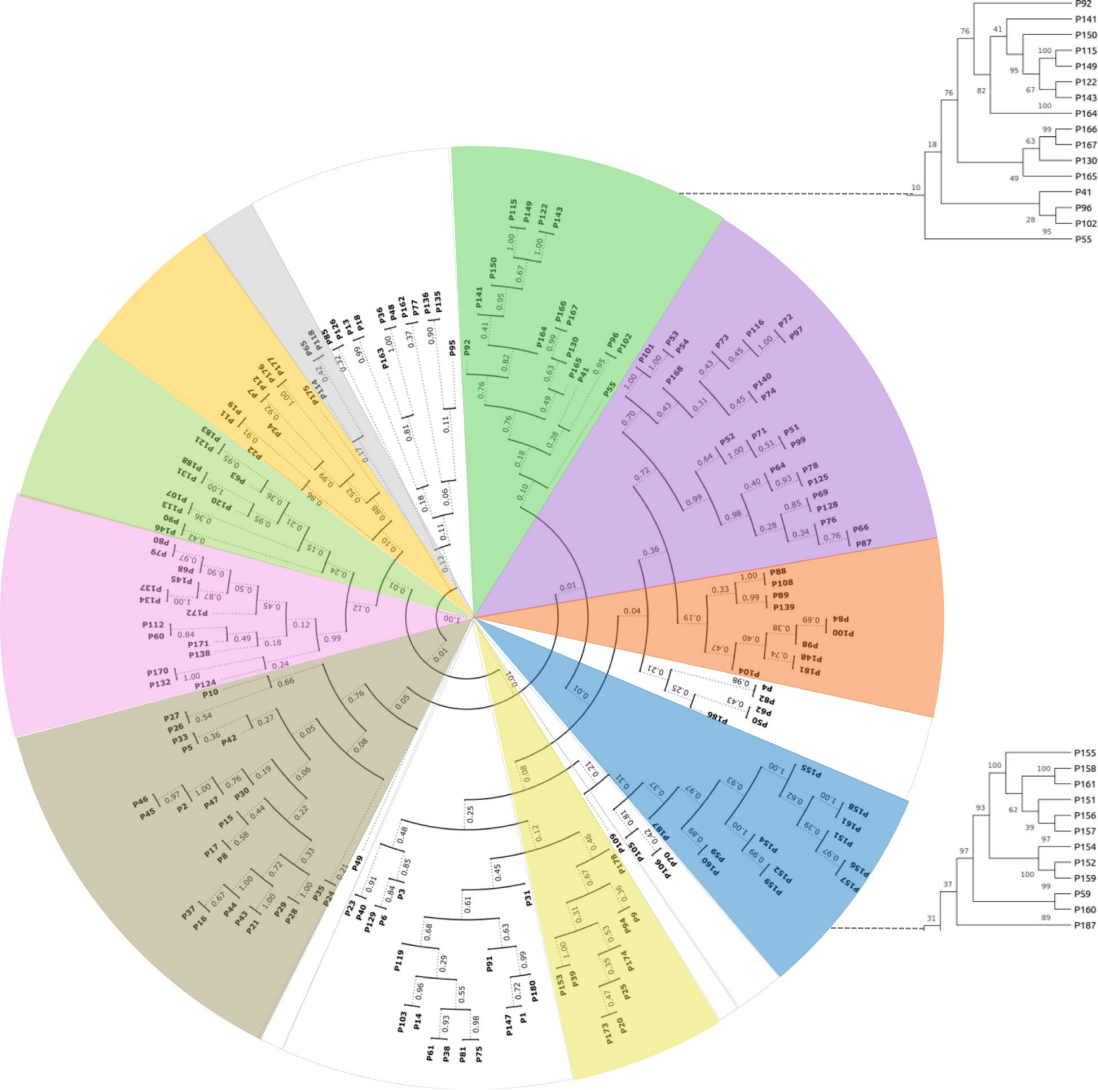
Phylogenetic tree of *R. opacus* R7 proteins containing the catalytic subunit of hydroxylases and monooxygenases. The tree was constructed aligning selected reference protein sequences with R7 sequences. Numbers represent the bootstrap values on the branches calculated for maximum likelihood (ML) method selected from the package MEGA version 6 with 100 bootstraps. Distinct clades are labeled with different colors: green, clade 1; purple, clade 2; red, clade 3; blue, clade 4; yellow, clade 5; brown, clade 6; pink, clade 7; light green, clade 8; orange, clade 9; grey, clade 10. White clades surrounded by grey lines correspond to not classified proteins: clade of not classified protein 1–6. The extended clade 1 includes the R7 proteins PheA1a, PheA2a, PheA3a; the extended clade 4 includes the R7 PrmA protein. The tree image was built with the ETE Toolkit using the circular plot function, with equal branch lengths and labeling each node with bootstrap support. Abbreviation of hydroxylase and monooxygenase names are reported in Additional file 2: Table S2

From all the sequences derived from the whole genome of R7, the attention was also focused on putative sequences annotated as monooxygenases/hydroxylases. Then, a multiple amino acidic sequence alignment and a clusterization analysis were performed to predict protein functions using characterized monooxygenases/hydroxylases from different bacteria as reference (Fig. 3). All the sequences are listed in Additional file 2: Table S2, including the reference sequences with the relative source strain and all the sequences belonging to the R7 strain. The sequences obtained by this analysis are clustered into 10 clades. Clade number 1 includes the phenol hydroxylases P164, P165, P166, P167, P149 and P150 used as reference sequences. Amongst these sequences, solely the P149 of *R. opacus* 1CP [26] was found to be similar to P115 (PheA1a) (98%), P122 (PheA2a) (92%), and P143 (PheA3a) (92%) of *R. opacus* R7. Accordingly, from this clade we selected the P115, P122 and P143 sequences (encoded by the *pheA1a* gene, *pheA2a* gene, and *pheA3a* gene, respectively) for further molecular analysis.

In clade number 4, only the P59 (PrmA) (encoded by the *prmA* gene) of R7, annotated as *alpha* chain methane monooxygenase component, is shown to cluster with all the phenol hydroxylases/monooxygenases used as reference sequences. Among the reference sequences, the TouA component (P151) of Toluene *o*-xylene Monooxygenase (ToMo) and the PhN component (P152) of Phenol Hydroxylase (PH) from *P. stutzeri* OX1, were mainly considered because they are the most described in literature for *o*-xylene oxidation [27–30]. In fact, comparing the amino acid sequences of the TouA component (P151) of ToMo, the PhN component (P152) of PH, and of PrmA (P59), the most residues of the catalytic site of the three proteins were found to be conserved. Thus, P59 was selected to investigate its involvement in *o*-xylene degradation.

R7 proteins of clades number 2, 3 and 5, 6 including cyclohexanone monooxygenases [31], FAD-dependent monooxygenases [32], salicylate hydroxylases [33], and putative P450 hydroxylases respectively [34], were excluded from further molecular analysis in this paper, as little is known regarding their putative involvement in *o*-xylene oxidation.

Regarding the other clades number 7, 8, 9, 10 including, respectively, putative nitrilotriacetate monooxygenases [35], alkanesulfonate monooxygenases [36], other putative monooxygenases/hydroxylases, and ubiquinone monooxygenases [37], we excluded their involvement in the hydroxylation/monooxygenation of *o*-xylene, because their function seems to be very distant from our search.

Moreover, the proteins of the non-classified clades 1–6 were excluded because they were lacking in reference sequences.

### Involvement of the *akb* genes in *o*-xylene degradation by RT-PCR experiments

**Fig. 4:**
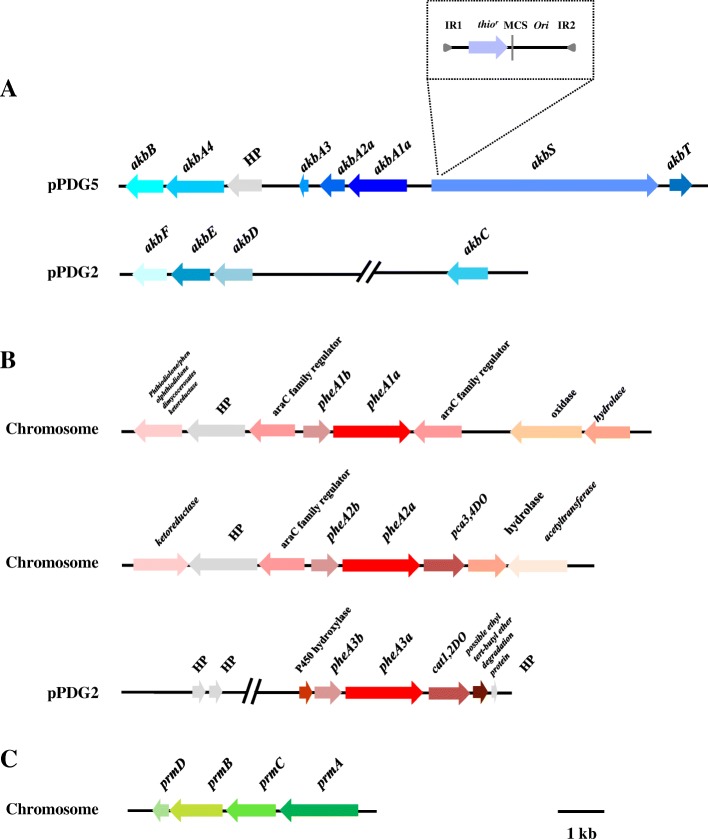
Genetic organization of the *akb, phe,* and *prm* genes in *R. opacus* R7. **a** On pPDG2 plasmid: *akbA1a,* large subunit of *o*-xylene dioxygenase; *akbA2a,* small subunit of *o*-xylene dioxygenase; *akbA3*, ferredoxin component; *akbA4*, reductase component; *akbB*, dihydrodiol dehydrogenase; *akbS*, sensor kinase; and *akbT*, response regulator. On pPDG5 plasmid: *akbC*, *meta*-cleavage dioxygenase, *akbD*, *meta*-cleavage hydrolase product, a*kbE*, hydratase and *akbF*, aldolase. Dashed box reported the IS*1415* insertion deriving from the transposon mutagenesis and dashed lines localized the insertion element within the *akbS* gene of R7. **b** Three *phe* gene clusters: *pheA1a* (P115), phenol hydroxylase, and *pheA1b*, phenol hydroxylase-reductase component located on the chromosome; *pheA2a* (P122), phenol hydroxylase, and *pheA2b*, phenol hydroxylase-reductase component located on the chromosome; *pheA3a* (P143), phenol hydroxylase, and *pheA3b*, phenol hydroxylase-reductase component located on the pPDG2 plasmid. **c** *prm* gene cluster located on the chromosome: *prmA* (P59), large hydroxylase subunit of a monooxygenase, *prmC*, small hydroxylase subunit of a monooxygenase, *prmB*, reductase component, and *prmD*, regulatory coupling protein. Genes with unknown or hypothetical functions were reported as HP. Identified genes (listed in Table 1) and their orientation are shown by arrow

Analyses of the R7 genome sequences evidenced the presence of the *akbA1a* gene in the *akb* gene cluster allocated on the megaplasmid pPDG5 (Table 1). This gene, coding for a large subunit dioxygenase component (AkbA1a), clustered with the following: the *akbA2a* gene coding for a small subunit dioxygenase component (AkbA2a), the *akbA3* gene for a ferredoxin component (AkbA3), the *HP* sequence for an hypothetical protein (HP) of unknown function, the *akbA4* gene for a reductase component (AkbA4), and the *akbB* gene coding for a dihydrodiol dehydrogenase (AkbB) (Fig. 4, panel a). Downstream (in the opposite direction) of these sequences, we found two sequences homologous (near the 80%) to the *akbS* and *akbT* sequences encoding for the sensor and regulator elements of DK17 strain, potentially involved in the regulatory mechanism.

**Table 1:** List of the identified genes

Gene name	Protein name	Homologous protein	Bacterium	aa Identity	Function	Accession number	Reference
*akbB* (813 bp)	AkbB	AkbB	*R.* sp. DK17	85%	Dihydrodiol dehydrogenase	AII11489.1	Kim et al., 2004
*akbA4* (1263 bp)	AkbA4	AkbA4	*R.* sp. DK17	81%	Ferredoxin reductase	AII11490.1	Kim et al., 2004
*akbA3* (150 bp)	AkbA3	AkbA3	*R.* sp. DK17	69%	Ethylbenzene dioxygenase ferredoxin	CP008952.1	Kim et al., 2004
*akbA2a* (549 bp)	AkbA2a	AkbA2a	*R.* sp. DK17	84%	Ethylbenzene dioxygenase small subunit	AII11492.1	Kim et al., 2004
*akbA1a* (1323 bp)	AkbA1a	AkbA1a	*R.* sp. DK17	92%	Ethylbenzene dioxygenase large subunit	AII11493.1	Kim et al., 2004
*akbS* (4812 bp)	AkbS	AkbS	*R.* sp. DK17	76%	Sensor kinase	AII11494.1	Kim et al., 2004
*akbT* (534 bp)	AkbT	AkbT	*R.* sp. DK17	86%	Response regulator	AII11495.1	Kim et al., 2004
*akbF* (762 bp)	AkbF	AkbF	*R.* sp. DK17	63%	4-Hydroxy-2-oxovalerate aldolase	AII11049.1	Kim et al., 2004
*akbE* (900 bp)	AkbE	AkbE	*R.* sp. DK17	64%	2-Hydroxypenta-2,4-dienoate hydratase	AII11050.1	Kim et al., 2004
*akbD* (858 bp)	AkbD	AkbD	*R.* sp. DK17	67%	2-Hydroxy-6-oxo-6-phenylhexa-2,4-dienoate hydrolase	AII11051.1	Kim et al., 2004
*akbC* (900 bp)	AkbC	AkbC	*R.* sp. DK17	87%	2,3-Dihydroxybiphenyl 1,2-dioxygenase	AII11058.1	Kim et al., 2004
*pheA1a* (1617 bp)	PheA1a	PheA1 (1) PheA1 (2) PheA1 (3)	*R. opacus* 1CP	83% 93% 98%	Phenol hydroxylase	AII08653.1	Gröning et al., 2014
*pheA1b* (573 bp)	PheA1b	PheA2 (1) PheA2 (2) PheA2 (3)	*R. opacus* 1CP	73% 100% 74%	Phenol hydroxylase reductase component	AII08654.1	Gröning et al., 2014
*pheA2a* (1617 bp)	PheA2a	PheA1 (1) PheA1 (2) PheA1 (3)	*R. opacus* 1CP	83% 100% 92%	Phenol hydroxylase	AII08806.1	Gröning et al., 2014
*pheA2b* (561 bp)	PheA2b	PheA2 (1) PheA2 (2) PheA2 (3)	*R. opacus* 1CP	69% 75% 94%	Phenol hydroxylase reductase component	AII08807.1	Gröning et al., 2014
*pheA3a* (1617 bp)	PheA3a	PheA1 (1) PheA1 (2) PheA1 (3)	*R. opacus* 1CP	83% 97% 91%	Phenol hydroxylase	AII10865.1	Gröning et al., 2014
*pheA3b* (654 bp)	PheA3b	PheA2 (1) PheA2 (2) PheA2 (3)	*R. opacus* 1CP	61% 64% 58%	Phenol hydroxylase reductase component	CP008949.1	Gröning et al., 2014
*prmA* (1635 bp)	PrmA	PrmA	*R. jostii* RHA1	97%	Methane monooxygenase component A *alpha* chain	AII03499.1	Sharp et al., 2007
*prmC* (1044 bp)	PrmC	PrmC	*R. jostii* RHA1	94%	Methane monooxygenase component C	AII03498.1	Sharp et al., 2007
*prmB* (1107 bp)	PrmB	PrmB	*R. jostii* RHA1	97%	Methane monooxygenase component A *beta* chain	AII03497.1	Sharp et al., 2007
*prmD* (342 bp)	PrmD	PrmD	*R. jostii* RHA1	98%	Methane monooxygenase regulatory protein	AII03496.1	Sharp et al., 2007

Moreover, we found a second group of genes (*akbCDEF* genes) coding for complete *meta*-cleavage enzymes of the lower pathway allocated on the pPDG2 plasmid, including: a *meta*-cleavage dioxygenase (AkbC), a *meta*-cleavage hydrolase (AkbD), an hydratase component (AkbE), and an aldolase (AkbF), respectively.

**Fig. 5:**
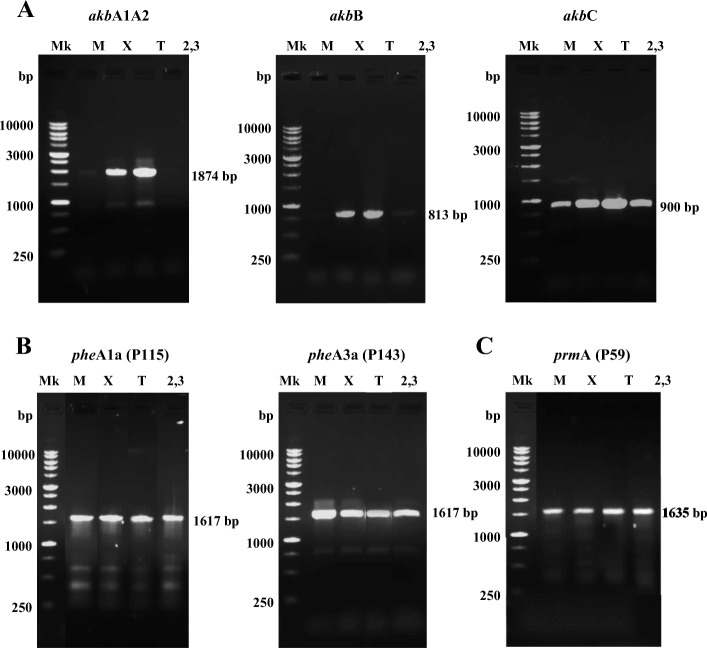
RT-PCR experiments from RNA extracted from *R. opacus* R7. RT-PCR experiments with *akbA1A2*, *akbB* and *akbC* genes (**a**); RT-PCR experiments with *pheA1a*, *pheA3a* (**b**), and with *prmA* genes (**c**). M 100 to 10,000-bp markers, M growth on mineral medium M9 and malate used as control, X growth on *o*-xylene, T growth on toluene, 2,3 growth on 2,3-dimethylphenol

The involvement of the *akb* genes in the *o*-xylene degradation of R7 strain was analyzed by RT-PCR experiments. For this, RT-PCR were performed with RNA derived from *R. opacus* R7 cells grown in presence of *o*-xylene, or 2,3-DMP, or toluene, or malate as control. Separate cDNA synthesis reactions were performed and cDNA was then amplified with primer pairs used to amplify the target genes. The target genes were *akbA1A2a* coding for the small and the large components of the dioxygenase, or *akbB* coding for the dihydrodiol dehydrogenase*,* or *akbC* coding for the *meta*-cleavage dioxygenase. RT-PCR analysis showed that the *akbA1A2a*, *akbB,* and *akbC* genes were transcribed in R7 cells after growth on *o*-xylene (or toluene) (Fig. 5, panel a). These results indicate that *o*-xylene induced the transcription of the *akb* genes, suggesting the involvement of a dioxygenation route for R7 *o*-xylene degradation. But, as described above, the analyses of intermediates revealed that 2,3-dimethylphenol and 3,4-dimethylphenol were non-inducers of the pathway. Indeed, RT-PCR experiments with the same *akbA1A2a*, *akbB,* and *akbC* genes, after growth in presence of 2,3-dimethylphenol and 3,4-dimethylphenol, did not show any amplification. This propelled us in the direction to search in the R7 genome for other sequences encoding for monooxygenases/hydroxylases and to demonstrate their subsequent involvement in alternative pathways for *o*-xylene degradation leading to dimethylphenols.

### Involvement of the *akb* genes in *o*-xylene degradation by the identification of *R. opacus* R7 mutants in this cluster

Random mutagenesis performed after electroporation of R7 cells with the pTNR vector generated mutants of R7 unable to growth on *o*-xylene.

**Fig. 6:**
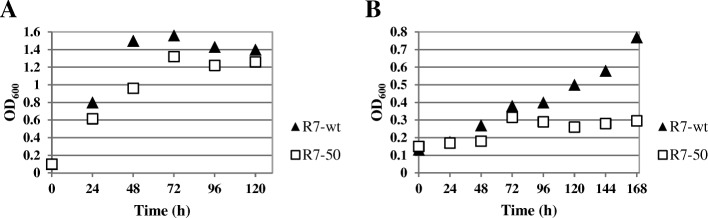
Growth curves on *o*-xylene in wild type *R. opacus* R7 and R7–50 mutant strain. Cells were grown in presence of M9 mineral medium and malate (**a**) or M9 mineral medium supplied in an atmosphere saturated with *o*-xylene (**b**)

We investigated the growth phenotypes and substrate transforming capabilities of R7 mutants by the transposon insertion detection. Among the single transposed mutant, the clearest phenotype was observed in the R7–50 mutant strain, in which the mutation was constituted by the insertion of the transposon in the *akbS* gene (Fig. 4, dashed box). This strain was considered a leaky mutant for the growth on *o*-xylene, as it is reported in Fig. 6 (panel a growth on malate, panel B growth on *o*-xylene) in comparison to the R7 wild type strain. In fact, Fig. 6 displays a lower rate of growth for the mutant in respect to the wild type strain when grown on *o*-xylene, while there is a similar trend when both strains are grown on malate.

These data are in accordance with what was observed by Kim et al. [38] when the ATP-binding motif of the sensor *akbS* gene was mutated in the DK17 strain. The mutation in the *akbS* gene allowed the incapacity of DK17 strain to grow well on *o*-xylene. So, our results indicate that *akbS* is necessary for the growth on *o*-xylene also in the R7 strain. Moreover, these data confirm the results of RT-PCR and suggest the kind of regulation involved in the *o*-xylene degradation pathway in the R7 strain. Indeed, this degradation process is likely mediated by the system sensor-regulator AkbS-AkbT through the binding of *o*-xylene.

### Identification of the involvement of the *phe* genes and the *prm* genes in *o*-xylene degradation by RT-PCR experiments

Based on the identified sequences from genome analysis and the previous metabolic intermediates of R7 *o*-xylene degradation pathway, we analyzed the involvement of some putative sequences encoding for monooxygenases/phenol hydroxylases in this pathway. In particular, we selected the sequences deriving from clade number 1 (called *phe* sequences) and the sequences from clade number 4 (called *prm* sequences) (Table 1). We identified a first *phe* sequence (*pheA1a*) encoding for the monooxygenase PheA1a (P115) that showed a nucleotide identity of 98% with the sequences of the *pheA1(3)* gene of *R. opacus* 1CP, involved in the phenol hydroxylation [26]. In the R7 genome, this gene (*pheA1a*) clustered with another gene (*pheA1b*) encoding for a phenol hydroxylase-reductase component and other open reading frames (ORFs) encoding for unknown functions (Fig. 4, panel b). From the same group of sequences, we also selected other two sequences, *pheA2a* (PheA2a) (P122) homologous to the *pheA1(2)* gene (99%) of *R. opacus* 1CP and *pheA3a* (PheA3a) (P143) homologous to the *pheA1(2)* gene (97%) of *R. opacus* 1CP. The *pheA2a* gene clustered with the *pheA2b* gene coding for a phenol hydroxylase-reductase component and a *pca* 3,4-dioxygenase; while the *pheA3a* gene clustered with the *pheA3b* gene coding for a phenol hydroxylase-reductase component and a catechol 1,2 dioxygenase. The last *phe* gene cluster was found allocated on the pPDG2 plasmid.

We performed RT-PCR experiments on these identified sequences after growth of R7 cells in presence of *o*-xylene, or toluene, or 2,3-DMP, or malate as control. Separate cDNA synthesis reactions were performed and cDNA was then amplified with primer pairs used to amplify the target genes. RT-PCR analysis showed that in R7 cells grown on *o*-xylene (or on toluene), both the *pheA1a* gene (P115) and the *pheA3a* gene (P143) were amplified as well as on malate (Fig. 5, panel b). Among the identified *phe* sequences, the *pheA2a* sequence (P122) was not tested because we decided to test only the main representatives *pheA1a* (P115) and *pheA3a* (P143) as they are allocated on the chromosome and on the pPDG2 plasmid, respectively.

Moreover, as R7 strain was also able to oxidize *o*-xylene leading to the corresponding 2-methylbenzylalcohol, and data on RHA1 strain indicated the presence of genes up-regulated on propane coding for components of ethylbenzene dioxygenase [39], we decided to include the *prm* genes in the analysis (Fig. 4, panel c). The *prm* genes were found in a cluster constituted by the *prmA,C,B,D* genes, allocated on the chromosome with a percentage of amino acid identity near the 90% with the corresponding gene products of RHA1 strain. The *prmA* gene and *prmC* gene coded the large hydroxylase and the small hydroxylase subunits of a monooxygenase (annotated as propane monooxygenase), as *prmB* for the reductase component and *prmD* for the regulatory coupling protein, respectively.

Also concerning these genes, we tested their involvement in *o*-xylene degradation by expression of the *prmA* gene (P59) in presence of the same substrates reported above for *phe* genes (Fig. 5, panel c). This gene was expressed in the presence of *o*-xylene as well as on malate, toluene and DMPs. These results indicated that the *prm* genes were amplified similarly to the *phe* genes*,* suggesting that they could work even when the strain was in absence of the hydrocarbon or phenols. Moreover, the gene redundancy of several monooxygenases/phenol hydroxylases supported the hypothesis of alternative pathways for *o*-xylene degradation in R7 strain. At the same time, the amplification of the PrmA (P59) could explain the formation of the corresponding 2-methylbenzyalcohol.

### Quantitative real-time RT-PCR (qPCR) analysis

Quantitative real-time reverse transcription-PCR (qPCR) experiments were performed to quantify the levels of transcription of *akbA1a* (AkbA1a), *prmA* (P59) and *pheA1a* (P115) genes of R7 strain, representative of the selected catalytic subunit of different oxygenase systems putatively involved in *o*-xylene oxidation. qPCR experiments were performed after growth of R7 cells in presence of *o*-xylene, toluene and 2,3-DMP or malate as control. The values of transcription after R7 malate-grown cells were used as a basal level for comparison with the quantities determined with the substrates of interest. The level of *akbA1a* gene was approximately 19 ± 7.5-fold higher after growth on *o*-xylene (with a similar trend on toluene) than on malate. On the other hand, this analysis confirms that *prmA* and *pheA1a* gene transcription levels increased much less, which probably reflects their constitutive expression. In fact, the transcription levels of *akbA1a* gene in respect to *prmA* and *pheA1a* genes after growth on *o*-xylene (and on toluene), were found to be significantly different, with respective values of 0.23 ± 0.04 and 0.44 ± 0.11 (Table 2). A different trend was observed for the expression of the *prmA* and the *pheA1a* genes (*akbA1a* gene was not tested as it was not amplified in RT-PCR) after growth on 2,3-DMP. In this case, results showed an increase of the *pheA1a* transcription levels 5.18 ± 0.91-fold higher after growth on the corresponding dimethylphenol, whereas *prmA* was not induced.

**Table 2:** qPCR analysis. Relative gene expression of *R. opacus* R7 grown on *o*-xylene, toluene and 2,3-dimethylphenol (2,3-DMP)

Substrate	Normalized genes amount relative to malate condition (2^-ΔΔCt^)					
	*akbA1a*		*prmA*		*pheA1a*	
*o*-xylene	19.14	±7.55	0.23	±0.04	0.44	±0.11
toluene	15.14	±6.56	1.53	±0.39	2.14	±0.65
2,3-DMP	–	–	0.04	±0.01	5.18	±0.91

Values are means of three replicates ± standard deviation

These results demonstrated that *o*-xylene was able to activate mainly the transcription of the *akbA1a* gene whilst a very low level of the other two genes during the aerobic growth of R7 cells on *o*-xylene. Meanwhile, in presence of 2,3-DMP a higher level of expression of phenol hydroxylase was observed.

### Involvement of the *prm* genes by cloning and expression of the activity in *R. erythropolis* AP

In order to evaluate the role of the *prmACBD* gene cluster in the *o*-xylene metabolism, the region of 4.3 kb was isolated from R7 genomic DNA as *Nde*I/*Hind*III fragment. The PCR product was cloned into the shuttle-vector *E. coli*-*Rhodococcus* pTipQC2 to obtain pTipQC2-*prmACBD-*R7.

**Fig. 7:**
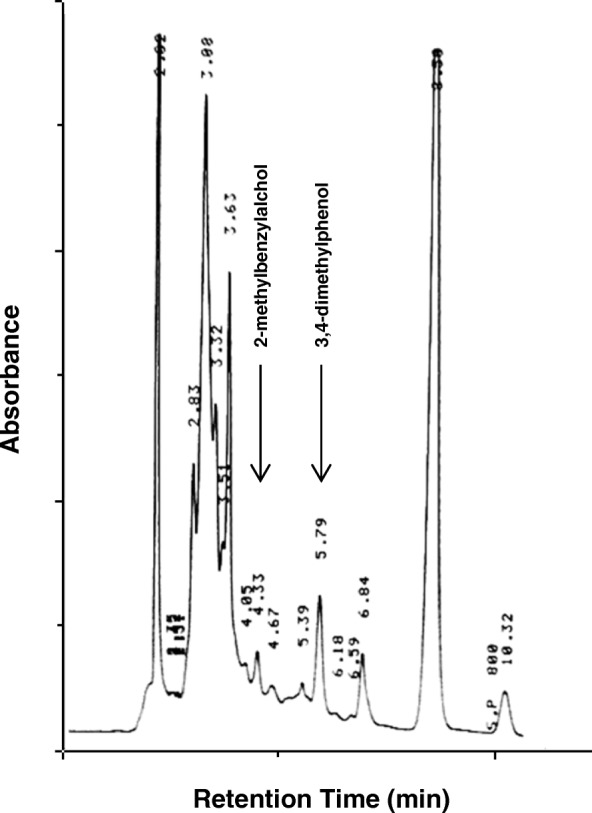
Reverse-Phase HPLC analysis of culture broth of *R. erythropolis* AP (pTipQC2-*prmACBD-*R7). The analysis was performed after exposition to *o*-xylene of the strain on the water phase of the biphasic system with isoctane. The retention times were 4.33 min for 2-methylbenzylalcohol, 5.79 min for 3,4-dimethylphenol

The recombinant plasmid pTipQC2-*prmACBD-*R7 was isolated from *E. coli* DH5a and transferred by electroporation into *Rhodococcus erythropolis* AP, which was not able to use the *o*-xylene as only carbon and energy source. The *prmACBD* gene cluster was expressed under the inducible thiostrepton promoter (P*tipA*) through experiments with resting cells of *R. erythropolis* AP (pTipQC2-*prmACBD-*R7) exposed to *o*-xylene to identify the metabolites. The activity of the recombinant strain was compared to the activity of wild type AP strain treated in the same conditions as control. *R. erythropolis* AP (pTipQC2-*prmACBD-*R7) cells, which were pre-grown on LB and washed in mineral medium M9, were exposed to *o*-xylene dissolved in isoctan in a biphasic system. The water phase was analyzed at different incubation times by reverse-HPLC analysis; 3,4-dimethylphenol and 2-methylbenzylalcohol were identified by comparison with reference compounds (standard mixture) (Fig. 7). These compounds were observed in the first 2 h of exposure, then they were progressively metabolized and disappeared after 6 h. It was not possible to confirm the formation of the 2,3-dimethylphenol. None of these metabolites was identified in the wild type host strain. These results suggested that the *prmACBD* gene cluster could have a role within the *o*-xylene metabolism, in particular in the first step of oxidation.

## Discussion

The genome-based analysis of *R. opacus* strain R7 revealed a considerable multiplicity of genes potentially involved in *o*-xylene catabolism. Although much is known about the ability of *Rhodococcus* strains to grow on toluene and ethylbenzene [39–41], little is known about the catabolism of *o*-xylene in bacteria belonging to the *Rhodococcus* genus [11]. *R. opacus* R7 is a strain isolated for its ability to grow on *o*-xylene as the only carbon and energy source. The strain is able to grow on *o*-xylene but not on *m*- and *p*-xylene. The inability of R7 strain to grow on the latter two compounds reinforced the hypothesis that the xylenes are metabolized at least by two different pathways [12]. Moreover, we have previously identified [22] the 2,3- and 3,4-dimethylphenols, as the main intermediates in the culture medium of R7 exposed to *o*-xylene, which are used by the strain as the only carbon and energy source, and not the corresponding dihydrodiol. Otherwise, in literature is reported by Kim et al. [10] that *o*-xylene is oxidized to the corresponding dihydrodiol. Moreover, Kim and co-authors also reported the direct formation of dimethylphenols in presence of *m*- and *p*-xylenes by *Rhodococcus* sp. strain DK17. This suggests that alternative oxidation mechanisms of xylenes are possible [11]. Whether this is through the action of a dioxygenase, forming a dihydrodiol, which dehydrates to a phenolic intermediate, or through the action of a monooxygenase which can directly hydroxylate the aromatic ring (or a combination of the two steps), it remains to be investigated.

**Fig. 8:**
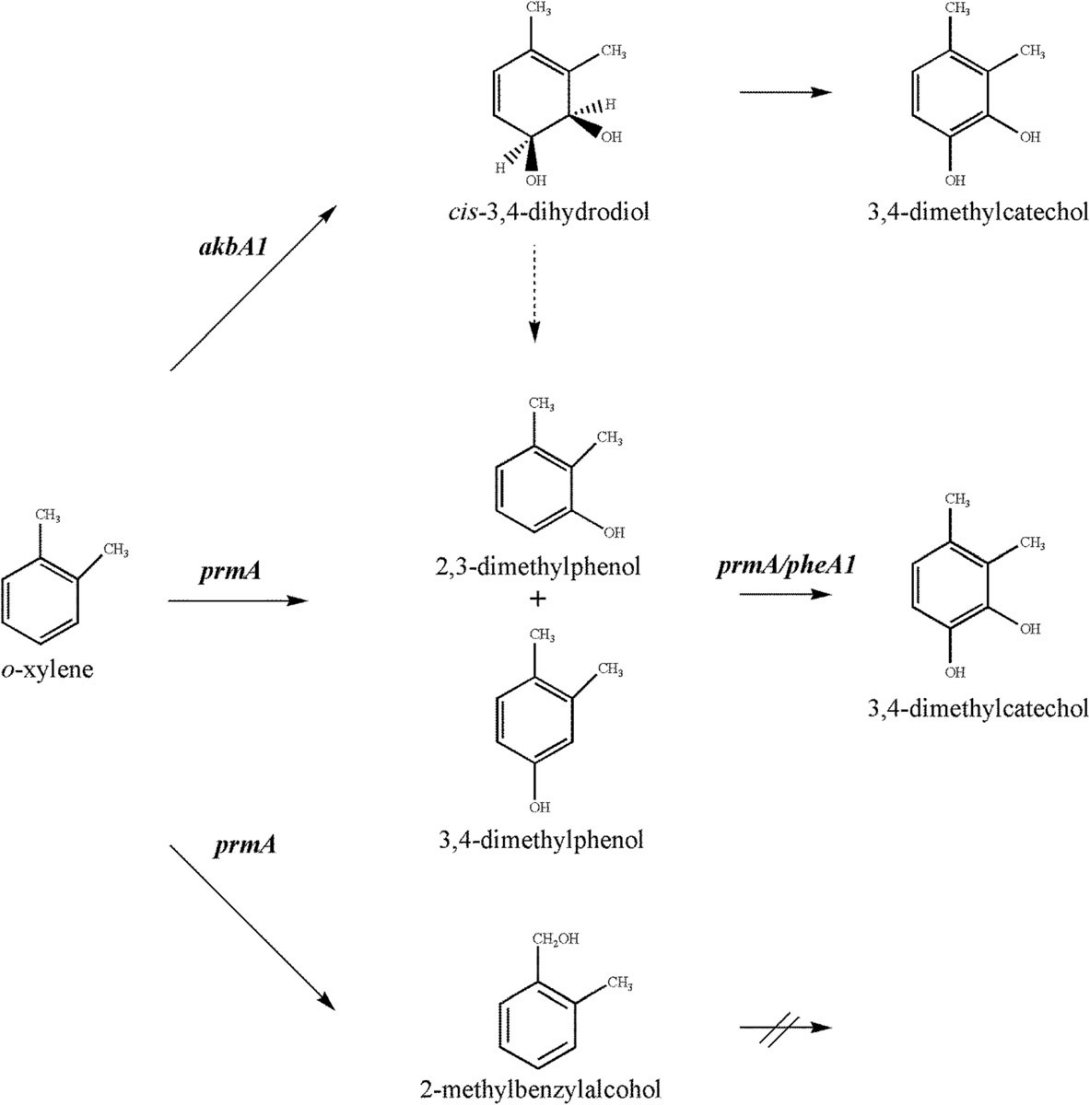
Proposed metabolic pathways involved in *o*-xylene degradation in *R. opacus* R7. *akbA1, o*-xylene dioxygenase route; *prmA,* monooxygenase route; *pheA1*, phenol hydroxylase route. Predicted genes are listed in Table 1. Dashed arrow indicates a spontaneous dehydration

In this context, a genome-based approach was used to better understand the R7 peculiar *o*-xylene pathway. Consequently, we decided to investigate the role of some selected genes and to demonstrate their involvement in this catabolism (Fig. 8). As a first step we analyzed and clustered all the R7 genome oxygenase sequences generating two phylogenetic trees (Figs. 2 and 3).

From the dioxygenase tree analysis, we selected the AkbA1a dioxygenase coded by the *akbA1a* gene (included in the *akb* gene cluster), whose sequences are 90% homologous to the sequence of the DK17 strain. In this paper, we demonstrate that the *akb* genes are induced by the presence of *o*-xylene supplied as the only carbon and energy source, both by RT-PCR/qPCR experiments and by selection of the R7–50 leaky mutant on *o*-xylene. RT-PCR analysis showed that *o*-xylene activated the transcription of the *akbA1A2* genes coding for a *o*-xylene dioxygenase and the *akbB* gene coding for a dihydrodiol dehydrogenase, suggesting the dioxygenation route for the *o*-xylene oxidation. These data confirmed what was reported for DK17 strain [10].

However, these data are apparently in disagreement with what we observed in the R7 metabolic analysis [21], because we identified the 2,3- and 3,4-DMPs and no literature data supports enough that DMPs could derive from the dehydration of the corresponding dihydrodiol. Moreover, R7 strain was also able to grow on the corresponding 2,3- and 3,4-DMPs as the only carbon and energy source, suggesting an alternative pathway for the *o*-xylene oxidation through a monooxygenation. Since the focus of the present work was the identification of genes involved in the initial oxidation of *o*-xylene, we wanted to verify the formation mechanism of 2,3- and 3,4-DMPs from *o*-xylene. To support this hypothesis, we analyzed all the R7 monooxygenases/phenol hydroxylases sequences (Fig. 3). As a result, on the basis of sequence identities with other *o*-xylene monooxygenases/phenol hydroxylases, genome sequences of other bacteria, and comparison of the protein catalytic site, we found putative sequences that could be involved in *o*-xylene degradation. In clade number 4, only one R7 protein sequence (PrmA) (P59) showed a significant amino acid identity with respect to reference *o*-xylene monooxygenases (like Toluene *o*-xylene Monooxygenase, ToMo). As the ToMo is the best known monooxygenase able to oxidize *o*-xylene with the formation of the corresponding DMPs, we hypothesized that PrmA could also be involved in this monooxygenation/hydroxylation. Thus, we investigated the role of the *prmA* gene in the *o*-xylene (or toluene) degradation by RT-PCR and quantitative real time RT-PCR experiments on R7 cells grown in presence of *o*-xylene. Results showed that although the levels of the *prmA* gene increased little with respect to the growth on malate, it could be a first indication of the involvement of PrmA in this pathway. Then, the activity of the PrmACBD multicomponent monooxygenase was examined after the cloning of the corresponding genes in another *Rhodococcus* strain unable to use *o*-xylene like *R. erythropolis* AP. Results evidenced that *o*-xylene was slowly transformed in 3,4-DMP and 2-methylbenzylalcohol, that could be then metabolized by other monooxygenases/phenol hydroxylases. Indeed, R7 strain is able to grow also in presence of the DMPs when supplied as sole carbon and energy source. To support these data we have also looked for R7 monooxygenases/phenol hydroxylases. From R7 genome analysis, we identified three sets of two component phenol hydroxylases, constituted of an oxygenase component and a reductase component; two sets were allocated on the chromosome on two different regions, and the third one on the pPDG2 plasmid of R7, respectively. In this case, *phe* genes were selected for RT-PCR experiments to evaluate their involvement in *o*-xylene oxidation. In all the growth conditions utilized we observed an amplification of the corresponding genes. The *pheA1a* encoding for the PheA1a (P115) was also tested in the quantitative real time RT-PCR on R7 cells grown in presence of *o*-xylene, toluene, 2,3-DMP and malate. Results showed a significant increase of *pheA1a* gene transcriptional levels during the growth on 2,3-DMP in respect to the growth on malate. This suggested that the *phe* genes could be involved in the second step of *o*-xylene degradation.

R7 strain showed a substrate versatility in respect to different substituted phenols, including 2,3-DMP and 3,4-DMP. This substrate versatility could likely be the result of gene redundancy and the presence of several phenol hydroxylase (iso)enzymes. These data are in accordance with literature, where it is reported that: three phenol hydroxylases in *R. opacus* B4, four in *R. opacus* M213, five in *R. opacus* PD630 and four in the reference strain *R. jostii* RHA1 have evident activities and expression profiles for this class of enzymes in these bacteria [26].

Thus, considering such metabolic diversity of R7 strain, we would deduce that, although the *akb* genes are the specific activated genes for *o*-xylene degradation, other genes such as *prm* genes can induce an increase of levels of phenols that can converge towards the phenol oxidation route.

The co-activation of multiple oxygenases could contribute to such strategy in these kinds of bacteria particularly resistant to environmental stress. Indeed, it has been demonstrated [42] that large genome with multiple broad-specificity catabolic enzymes such as those reported in RHA1 strain could have a competitive advantage in environmental changing soil conditions.

## Conclusions

In conclusion, in this paper we demonstrate that *R. opacus* R7 is able to degrade *o*-xylene by the activation of the *akb* genes leading to the production of the corresponding dihydrodiol. Likewise, the redundancy of sequences encoding for several monooxygenases/phenol hydroxylases, supports the involvement of other genes that can induce an increase of levels of phenols that can converge towards the phenol oxidation route.

The activation of multiple converging oxygenase systems represents a strategy in bacteria of *Rhodococcus* genus to degrade a wide range of recalcitrant compounds and to persist in severely contaminated environments.

## Methods

### Bacterial strain and growth conditions

The bacterial strain used in this study is *R. opacus* R7, isolated for its ability to grow on naphthalene and *o*-xylene as previously described [21] (deposited to the Institute Pasteur Collection, CIP identification number 107348). The strain was grown at 30 °C in M9 mineral medium [43], supplemented with the following carbon sources as only carbon and energy source: *o*-xylene, toluene (final concentration of 1 g/l) or 2,3-dimethylphenol, 3,4-dimethylphenol (final concentration of 5–10 mM), or 2-dimethylbenzylalcohol, 3-dimethylbenzylalcohol or malate (final concentration of 10 mM). The *R. opacus* R7 growth on *o*-xylene, toluene, took place on M9 mineral medium in an atmosphere saturated with these aromatic compounds in a sealed system. The mutant R7–50 strain used in this paper was grown in the same conditions utilized for the wild type R7 strain.

*Rhodococcus erythropolis* AP, isolated in our laboratory (CIP 110799) for its ability to grow on diesel fuel, was maintained on M9 mineral medium in a saturated atmosphere of diesel fuel at 30 °C.

### Bioinformatic analysis: Nucleotide sequence determination and protein sequence analysis

The preliminary annotation of *R. opacus* R7 genome sequences was performed using the RAST (Rapid Annotation using Subsystem Technology) service [44].

BLASTn tool [45] of NCBI pipeline was used to determine nucleotide sequence homology and to make manual curation.

*R. opacus* R7 putative gene clusters for *o*-xylene catabolism were identified on chromosome and megaplasmids using BLAST tool and Clustal Omega [46].

*R. opacus* R7 protein sequences were preliminary annotated using the RAST that allowed to identify potential monooxygenases/hydroxylases and dioxygenases using text string searching.

These sequences annotated as monooxygenases/hydroxylases and dioxygenases were aligned separately against PDB (RCSB Protein Data Bank) database to identify reference sequences. Reference proteins were selected on the basis of the highest similarity or literature data. If no match was identify against PDB database, the same procedure was applied using BLASTp of NCBI pipeline.

Afterwards, the identified reference sequences were aligned against R7 genome using the NCBI pipeline in order to verify to have considered all R7 putative monooxygenases/hydroxylases and dioxygenases.

The retrieved sequences were aligned using the multiple sequence alignment (MSA) tool of Clustal Omega program using the default parameters (neighbour joining method, the Gonnet transition matrix, gap opening penalty of 6 bits, maintain gaps with an extension of 1 bit, used bed-like clustering during subsequent iterations, and zero number of combined iterations).

For each group of oxygenases, the MSA was used for the cluster analysis inferred using the maximum likelihood (ML) method selected from the package MEGA version 6 [47]. The following parameters were used: JTT matrix, used all sites and gamma distribution of mutation rates with gamma optimized to 2. As a test of inferred phylogeny, 100 bootstrap replicates were used.

The resulting groups allowed to define two different trees, one for all the dioxygenases and one for all the monooxygenases/hydroxylases of R7 showing clades with putative functions identified by InterPro/UniProt databases.

### Preparation, analysis, and DNA manipulation

Total DNA from *R. opacus* R7 was extracted according to method reported by Di Gennaro et al. [22]. The extract was precipitated by 0.1 volume of 3 M sodium acetate and after centrifugation, the DNA was isolated and purified. Standard methods of DNA manipulation were used in this work [43]. For the recovery and purification of DNA fragments from agarose, Extraction Kits by Nachery and Nagel (Fisher Scientific, Germany) were used. Amplification of fragment containing genes target was achieved by PCR performed using primers designed ad hoc (Additional file 3: Table S3 and Additional file 4: Table S4) to amplify the sequences of interest.

### RNA extraction and RT-PCR, quantitative real-time RT-PCR (qPCR)

Total RNA was extracted from bacterial cultures of *R. opacus* R7 (100 ml) grown at 30 °C on M9 mineral medium supplemented with different substrates supplied (as described above) as the only carbon and energy source: *o*-xylene, toluene at the concentration of 1 g/l, 2,3-dimethylphenol and 3,4-dimethylphenol at the concentration of 5–10 mM and 10 mM malate used as reference.

RNA extraction protocol was performed using the RNA-Total RNA Mobio Isolation Kit (Qiagen Italia, Italy) according to the manufacturer’s instructions and at the end the DNase treatment was performed. Reverse transcription was performed with iScript cDNA Synthesis kit (BIO-RAD, Italy) to obtain the corresponding cDNAs. For the cDNA synthesis 200 ng of total RNA was reverse-transcribed as follows: after denaturation for 5 min at 25 °C, reverse transcription was performed for 1 h at 42 °C and then 5 min of elongation at 85 °C.

RT-PCR experiments were performed by amplification of the cDNA samples, each in 25-μl PCR volume containing 2 μl of the reverse-transcribed RNA samples.

Amplifications of the *akbA1A2*, *akbB*, *akbC*, *prmA* (P59), *pheA1a* (P115), *pheA3a* (P143) genes and 16S rDNA were performed using 2.5 U/μl of Long Range DNA Rabbit Polymerase (Eppendorf, Germany).

Thermo cycling conditions were as follows: 3 min at 95 °C, 95 °C for 30 s, specific T_m_ for 45 s, 72 °C for 4 min, for 35 cycles; and 72 °C for 3 min. Amplification of 16S rDNA was performed using the universal bacterial primers 27f and 1495r as described in Di Gennaro et al. [21]. The internal housekeeping gene (16S rDNA) was used as reference to evaluate relative differences in the integrity of individual RNA samples.

Quantitative real-time Reverse Transcriptase-PCR (qPCR) analyses were performed on the same samples used for RT-PCR. The reverse-transcribed samples were amplified using the StepOnePlus Real-Time PCR System (Applied Biosystem, Italy). Each 10-μl qPCR volume contained 4.4 μl of the reverse-transcribed RNA samples, 5 μl of PowerUp SYBR Green Master Mix (Applied Biosystem, Thermo Scientific, Italy), and 300 nM of each primer, listed in Additional file 3: Table S3. Thermocycling conditions were as follows: 30 s at 95 °C, followed by 40 cycles of 5 s at 95 °C, 10 s at 60 °C and 45 s at 72 °C and one cycle 15 s at 95 °C, 1 min at 60 °C and 15 s at 60 °C. Expression of the housekeeping gene, 16S rDNA, was used as reference gene to normalize tested genes in *R. opacus* R7. The ΔΔCt method with 16S rDNA as reference gene was used to determine relative abundance of target transcripts in respect to malate as control. Data are expressed as mean ± standard deviation derived from at least three independent experiments.

In order to exclude DNA contamination, negative controls were performed by omitting the reverse transcriptase in RT-PCR experiments, which were conducted with the same temperature program and the same primer sets for 35 cycles of amplification.

The primers used in the RT-PCR analysis and qPCR are described in Additional file 3: Table S3.

### Mutant preparation

#### Transposon-induced mutagenesis in *R. opacus* R7 using IS1415 (pTNR-TA vector)

Plasmid pTNR-TA [48] was transferred into *R. opacus* strain R7 by electroporation as described by Treadway et al. [49], using a Gene Pulser II (BIO-RAD, Italy) set at 2.50 kV, 600 Ω, 25 μF in presence of maximum 1 μg DNA in a 2 mm-gap electro-cuvette (BIO-RAD, Italy). Afterwards, the electroporation mixture was suspended in 2.5 ml LB and it was incubated for 4 h at 30 °C under shaking. Cells were plated on Luria-Bertani (LB) supplemented with 12.5 μg/ml thiostrepton and they were grown at 30 °C for 5 days to select thiostrepton-resistant cells. Transposon-induced mutants were transferred to M9 mineral medium agar plates with 12.5 μg/ml thiostrepton and 10 mM malate.

The transposon-induced mutants obtained were tested on M9 mineral medium agar plates with the following carbon sources as the only carbon and energy source at the final concentration of 1 g/l: *o*-xylene; toluene; 2,3-dimethylphenol and 3,4-dimethylphenol (5–10 mM); 2 dimethylbenzylalcohol.

#### Analysis of pTNR-TA insertion sites

The genomic DNA of each transposon-induced mutant was extracted and the Two-Step gene walking PCR method was applied [50]. Insertions of IS1415 into the genomes of these mutants were confirmed by PCR using primers reported in Additional file 4: Table S4. Genomic DNA of the wild type strain was used as a negative control. Homology searches of the interrupted DNA sequences from mutants were conducted by BLAST (http://blast.ncbi.nlm.nih.gov/Blast.cgi) [45].

### Construction of the recombinant strain *R. erythropolis* AP (pTipQC2-*prmACBD*-R7)

The *prmACBD* gene cluster was ligated as *Nde*I/*Hind*III fragment into a shuttle-vector *E. coli*-*Rhodococcus*, pTipQC2 [51]. The ligation mixture was used to transform *E. coli* DH5α by electroporation with standard procedures [52] and the recombinant clones were selected on LB agar supplemented with ampicillin (100 μg/ml) at 37 °C. Ampicillin-resistant clones were selected and the recombinant plasmid (pTipQC2-*prmACBD-*R7) was isolated. The same recombinant plasmid was used to transform *R. erythropolis* strain AP by electroporation according to Zampolli et al. [23]. Immediately after electroporation, 2.5 ml recovery broth (LB medium with 1.8% sucrose) were added and cells were incubated at 30 °C for 4 h. Cells were plated on LB supplemented with chloramphenicol 50 μg/ml and grown at 30 °C for 3–4 days. Recombinant strain *R. erythropolis* AP (pTipQC2-*prmACBD-*R7) was used for bioconversion experiments in presence of *o*-xylene to evaluate the activity of the *prmACBD* system in comparison to the activity of the wild type strain.

### Bioconversion experiments of the recombinant strain *R. erythropolis* AP (pTipQC2-*prmACBD*-R7) in presence of *o*-xylene

Cells of *R. erythropolis* AP (pTipQC2-*prmACBD-*R7) were grown in 500-mL Erlenmeyer flasks containing 100 mL of LB at 30 °C. When the culture reached the O.D._600_ of 0.6, they were induced with thiostrepton. After overnight growth, the cells were collected by centrifugation for 10 min at 20,000 g, washed and re-suspended in the mineral medium M9 to have final O.D._600_ of 1.

Bioconversion experiments were performed in a biphasic system with *o*-xylene (1 g/l) dissolved in isoctan at 20% of the final volume of the culture. At fixed times, 2 h, 4 h, 6 h, and 24 h, a flask was sacrificed to determine the metabolites produced during growth on *o*-xylene. It occurred by HPLC analysis after acidification with H_2_SO_4_. After settling of the suspension, the aqueous phase was drawn from the organic phase, stripped under a gentle stream of nitrogen, resuspended in order to concentrate each culture 1:20 and filtered with 0.45 μm filters for HPLC analysis.

### Analytical methods

HPLC analyses were performed with a Waters 600E delivery system (Waters Corporation, Milford, MA, USA) equipped with a Waters 486 UV-Vis detector and a Waters reverse-phase μBondapak 3.9 × 300 mm C18 column. The mobile phase was acetonitrile:water (50:50) at a flow rate of 1 mL/min in isocratic conditions. The detection was carried out at 254 nm and the retention times of the identified peaks were compared with those of authentic compounds. Co-elution experiments were also performed in which the culture broth samples were supplemented with authentic compounds. In these conditions, the retention times were 4.33 min for 2-methylbenzylalcohol, 6.39 min for 2,3-dimethylphenol, and 5.79 min for 3,4-dimethylphenol.

## Additional files

Additional file 1: **Table S1.** Sequences used to generate dioxygenase tree. (DOCX 25 kb)

Additional file 2: **Table S2.** Sequences used to generate monooxygenase tree. (DOCX 44 kb)

Additional file 3: **Table S3.** List of utilized oligos. (DOCX 15 kb)

Additional file 4: **Table S4.** List of utilized oligos for transposon identification. (DOCX 13 kb)

## Abbreviations

2,3-DMP: 2,3-dimethyl phenol
3,4-DMP: 3,4-dimethyl phenol
BLAST: Basic Local Alignment Search Tool
BTEX: benzene, toluene, ethylbenzene and xylenes
cDNA: Complementary Deoxyribonucleic Acid
DMPs: dimethyl phenols
HP: hypothetical protein
HPLC: High-Performance Liquid Chromatography
LB: Luria-Bertani
NCBI: National Center for Biotechnology Information
O.D.600: Optical Density at 600 nm
ORFs: Open Reading Frames
PAHs: polycyclic aromatic hydrocarbons
PCBs: polychlorinated biphenyls
PDB: RCSB Protein Data Bank
qPCR: quantitative real time RT-PCR
RAST: Rapid Annotation using Subsystem Technology
RT-PCR: Reverse Transcriptase Polymerase Chain Reaction
ToMo/PH: Toluene *o*-xylene Monooxygenase/Phenol Hydroxylase
