# Supplementary material for: Genome-based analysis for the identification of genes involved in o-xylene degradation in Rhodococcus opacus R7
Source: BMC Genomics. 2018 Aug 6;19:587. doi: 10.1186/s12864-018-4965-6 (PMC6080516; doi:10.1186/s12864-018-4965-6)
Supplement: Supplementary file 1 — Table S1. Sequences used to generate dioxygenase tree. (DOCX 25 kb) [file 12864_2018_4965_MOESM1_ESM.docx]

| **Protein** | | **Location on Genome** | **Function** | **Accession Number** |
| --- | --- | --- | --- | --- |
| **C 1** | GenH |  | Gentisate 1,2-dioxygenase - *R*. *opacus* CIR2 | BAD35142.1 |
|  | GenH3 | pPDG1 | Gentisate 1,2-dioxygenase (EC 1.13.11.4) | AII10780.1 |
|  | GenH4 | pPDG4 | Gentisate 1,2-dioxygenase (EC 1.13.11.4) | AII11451.1 |
|  | GenH2 | chromosome | Gentisate 1,2-dioxygenase (EC 1.13.11.4) | AII09311.1 |
|  | GenH1 | chromosome | Gentisate 1,2-dioxygenase (EC 1.13.11.4) | AII05708.1 |
|  | DO2 | chromosome | Putative dioxygenase | AII08015.1 |
|  | TPHDO | chromosome | Tryptophan 2,3-dioxygenase  (EC 1.13.11.11) | AII09387.1 |
|  | TPHDO |  | Tryptophan 2,3-dioxygenase  (EC 1.13.11.11) - *R*. *opacus* PD630 | EHI40897.1 |
|  | DHpCD | chromosome | 2,3-Dihydroxy-*p*-cumate dioxygenase | AII08480.1 |
|  | DHpCD |  | 2,3-Dihydroxy *p*-cumate dioxygenase -  *R*. *opacus* B4 | BAH52889.1 |
|  | CatA2 | chromosome | Probable catechol 1,2-dioxygenase | AII07174.1 |
|  | Pca | chromosome | Protocatechaute dioxygenase | AII05947.1 |
|  | LSD | chromosome | Carotenoid oxygenase | AII05595.1 |
|  | LSD |  | Carotenoid oxygenase - *R*. *jostii* RHA1 | WP_054247633.1 |
|  | 2.3QD | chromosome | Quercetin 2,3-dioxygenase  (EC 1.13.11.24) | AII07182.1 |
|  | 2.3QD |  | Quercetin 2,3-dioxygenase  (EC 1.13.11.24) - *R*. *jostii* RHA1 | ABG95706.1 |
|  | HmgA | chromosome | Homogentisate 1,2-dioxygenase  (EC 1.13.11.5) | AII08874.1 |
|  | HmgA |  | Homogentisate 1,2-dioxygenase  (EC 1.13.11.5) - *R*. *jostii* RHA1 | WP_073365188.1 |
| **C 2** | 1.2-HQD | chromosome | Hydroxyquinol 1,2-dioxygenase | AII05734.1 |
|  | 1.2-HQD |  | Hydroxyquinol 1,2-dioxygenase - *R*. sp. M8 | WP_072634577.1 |
|  | 1.2-CHQDO | chromosome | 6-Chlorohydroxyquinol-1,2-dioxygenase | AII09320.1 |
|  | CatA1 | chromosome | Catechol 1,2-dioxygenase (EC 1.13.11.1) | CP008947.1 |
|  | DO3 | chromosome | Intradiol ring-cleavagedioxygenase | AII08811.1 |
|  | CatA4 | pPDG2 | Catechol 1,2-dioxygenase (EC 1.13.11.1) | AII10864.1 |
|  | CatA |  | Catechol 1,2-Dioxygenase - *R*. *opacus* 1CP | CAA67941.1 |
|  | CatA3 | chromosome | Catechol 1,2-dioxygenase (EC 1.13.11.1) | AII08813.1 |
| **C 3** | EtbAa1 |  | Ethylbenzene dioxygenase *alpha* subunit -  *R*. *jostii* RHA1 | BAC92712.1 |
|  | AkbA1a |  | Alkylbenzene dioxygenase - *R*. sp. DK17 | AAR90131.2 |
|  | AkbA1a | pPDG5 | Ethylbenzene dioxygenase large subunit | AII11493.1 |
|  | TDO |  | Toluene dioxygenase *alpha* subunit -  *P*. *putida* F1 | ABQ79012.1 |
|  | BphAa |  | Biphenyl 2,3-dioxygenase - *R*. *jostii* RHA1 | ABG99107.1 |
|  | PhtA | pPDG2 | Phthalate 3,4-dioxygenase *alpha* subunit | AII10987.1 |
|  | NarAa | pPDG4 | Biphenyl dioxygenase *alpha* subunit  (EC 1.14.12.18) | AII11432.1 |
| **C 4** | Rieske DO2 | chromosome | Phenylpropionate dioxygenase and related ring-hydroxylating dioxygenases, large terminal subunit | AII09375.1 |
|  | DO4 | pPDG1 | Putative dioxygenase *alpha* subunit YeaW | AII10625.1 |
|  | Rieske DO1 | chromosome | Ring hydroxylating dioxygenase, *alpha* subunit/Rieske (2Fe-2S) protein  (EC 1.14.12.18) | AII08355.1 |
|  | DO5 | pPDG2 | Putative dioxygenase hydroxylase component | AII10950.1 |
|  | BenDO |  | Benzoate 1,2-dioxygenase *alpha* subunit- *R*. *jostii* RHA1 | ABG94189.1 |
|  | BenDO2 | chromosome | Benzoate 1,2-dioxygenase *alpha* subunit (EC 1.14.12.10) | AII08802.1 |
|  | CutAb | chromosome | *p*-Cumatedioxygenase | AII08474.1 |
|  | CutAb |  | *p*-Cumate dioxygenase - *R*. *opacus* B4 | BAH52881.1 |
| **C 5** | 2NPD2 | chromosome | 2-Nitropropane dioxygenase | AII07138.1 |
|  | 2NPD4 | chromosome | 2-Nitropropane dioxygenase | AII08898.1 |
|  | 2NPD3 | chromosome | 2-Nitropropane dioxygenase NPD | AII08140.1 |
|  | 2NPD1 | chromosome | 2-Nitropropane dioxygenase  (EC 1.13.11.32) | AII06420.1 |
|  | 2NPD5 | chromosome | 2-Nitropropane dioxygenase  (EC 1.13.11.32) | AII10318.1 |
|  | 2NPD |  | 2-Nitropropane dioxygenase - *R*. *jostii* RHA1 | ABG92647.1 |
| **C 6** | TauD8 | chromosome | Taurine dioxygenase | AII09447.1 |
|  | TauD9 | chromosome | Taurine dioxygenase | CP008947.1 |
|  | TauD7 | chromosome | Taurine dioxygenase | AII09095.1 |
|  | TauD |  | Taurine dioxygenase-dependent sulfonate dioxygenase - *P*. *putida* KT2440 | NP_742398.1 |
|  | TauD3 | chromosome | Taurine dioxygenase | AII05751.1 |
|  | TauD4 | chromosome | Taurine dioxygenase | AII07329.1 |
|  | TauD6 | chromosome | Taurine dioxygenase | AII08691.1 |
|  | TauD5 | chromosome | Taurine dioxygenase | AII07522.1 |
|  | TauD1 | chromosome | Taurine dioxygenase | AII03182.1 |
|  | TauD2 | chromosome | Taurine dioxygenase | AII03183.1 |
| **C 7** | MhpB | pPDG2 | 3-Carboxyethyl catechol 2,3-dioxygenase (EC 1.13.11.16) | AII11046.1 |
|  | MhpB |  | 3-Carboxyethyl catechol 2,3-dioxygenase -  *R*. sp. DK17 | WP_029538291.1 |
|  | HPPD3 | chromosome | 4-Hydroxyphenylpyruvate dioxygenase (EC 1.13.11.27) | AII09141.1 |
|  | HPPD |  | 4-Hydroxyphenylpyruvate dioxygenase (EC 1.13.11.27) - *R*. *jostii* RHA1 | WP_054245951.1 |
|  | HPPD1 | chromosome | 4-Hydroxyphenylpyruvate dioxygenase (EC 1.13.11.27) | AII05993.1 |
|  | HPPD2 | chromosome | 4-Hydroxyphenylpyruvate dioxygenase (EC 1.13.11.27) | AII07999.1 |
| **C 8** | Cat 2.3-DO |  | Catechol 2,3-dioxygenase (EC 1.13.11.2) -  *R*. *opacus* PD630 | EHI47566.1 |
|  | Cat 2.3-DO | chromosome | Catechol 2,3-dioxygenase (EC 1.13.11.2) | AII07256.1 |
|  | Cat 2.3-DO3 | pPDG5 | Catechol 2,3-dioxygenase (EC 1.13.11.2) | AII11500.1 |
|  | DO1 | chromosome | 2,3-Dihydroxybiphenyl 1,2-dioxygenase (EC 1.13.11.39) | AII06164.1 |
|  | Cat 2.3-DO2 | pPDG2 | Catechol 2,3-dioxygenase (EC 1.13.11.2) | AII11025.1 |
|  | DHBD1 | chromosome | 2,3-Dihydroxybiphenyl 1,2-dioxygenase | AII04187.1 |
|  | BphDO | chromosome | Biphenyl-2,3-diol 1,2-dioxygenase 2  (EC 1.13.11.39) (Biphenyl-2,3-diol 1,2-dioxygenase II) (23OHBP oxygenase II) (2,3-dihydroxybiphenyl dioxygenase II) (DHBD II) | AII03287.1 |
|  | BphDO |  | Biphenyl 2,3-dioxygenase - *R*. *opacus* B4 | BAH52868.1 |
|  | PcaG | chromosome | Protocatechuate 3,4-dioxygenase *alpha* chain (EC 1.13.11.3) | AII09801.1 |
|  | PcaG |  | Protocatechuate 3,4-dioxygenase *alpha* chain (EC 1.13.11.3) - *R*. *opacus* 1CP | ANS29566.1 |
|  | DHBD5 | chromosome | 2,3-Dihydroxybiphenyl 1,2-dioxygenase | CP008947.1 |
|  | AkbC | pPDG2 | 1,2-Dihydroxynaphthalene dioxygenase | AII11058.1 |
|  | DHBD3 | chromosome | 2,3-Dihydroxybiphenyl 1,2-dioxygenase (EC 1.13.11.39) | AII06551.1 |
|  | DHBD |  | 2,3-Dihydroxybiphenyl dioxygenase –  *R*. *opacus* PD630 | AHK28227.1 |
|  | DHBD2 | chromosome | 2,3-Dihydroxybiphenyl 1,2-dioxygenase | AII05250.1 |
|  | DHBD4 | chromosome | 2,3-Dihydroxybiphenyl 1,2-dioxygenase  (EC 1.13.11.39) | AII08680.1 |
