# Supplementary material for: Genome-based analysis for the identification of genes involved in o-xylene degradation in Rhodococcus opacus R7
Source: BMC Genomics. 2018 Aug 6;19:587. doi: 10.1186/s12864-018-4965-6 (PMC6080516; doi:10.1186/s12864-018-4965-6)
Supplement: Supplementary file 2 — Table S2. Sequences used to generate monooxygenase tree. (DOCX 44 kb) [file 12864_2018_4965_MOESM2_ESM.docx]

| **Protein** | | | **Location on Genome** | **Function** | **Accession Number** |
| --- | --- | --- | --- | --- | --- |
| **C 1 – Phenol Hydroxylases and similar enzymes** | P92 | HpaB1 | chromosome | 4-Hydroxyphenylacetate 3-monooxygenase  (EC 1.14.13.2) | AII07219.1 |
|  | P141 | HpaB2 | pPDG2 | 4-Hydroxyphenylacetate 3-monooxygenase  (EC 1.14.13.2) | CP008949.1 |
|  | P150 | PheA1 |  | Phenol hydroxylase -  *Rhodococcus erythropolis* UPV-1 | ABS30825.1 |
|  | P115 | PheA1a | chromosome | 4-Hydroxyphenylacetate 3-monooxygenase  (EC 1.14.13.2) | AII08653.1 |
|  | P149 | PheA1 |  | (Chloro)Phenol hydroxylase -  *Rhodococcus opacus* 1CP | CAT00453.1 |
|  | P122 | PheA2a | chromosome | 4-Hydroxyphenylacetate 3-monooxygenase  (EC 1.14.13.2) | AII08806.1 |
|  | P143 | PheA3a | pPDG2 | 4-Hydroxyphenylacetate 3-monooxygenase  (EC 1.14.13.2) | AII10865.1 |
|  | P164 | SgcC |  | 4OO2 Chlorophenol-4-monooxygenase- *Streptomyces globisporus* | AAL06674.1 |
|  | P166 | TftD |  | 4G5E 2,4,6-Trichlorophenol 4-monooxygenase -  *Cupriavidus pinatubonensis* | AAM55214.1 |
|  | P167 | TftD |  | 3HWC Chlorophenol 4-monooxygenase - *Burkholderia cepacia* AC1100 | AAC23548.2 |
|  | P130 | HpaB3 | chromosome | 4-Hydroxyphenylacetate 3-monooxygenase  (EC 1.14.13.2) | AII09405.1 |
|  | P165 | HpaB |  | 2YYG 4-Hydroxyphenylacetate 3-monooxygenase - *Thermus thermophilus* | YP_144226.1 |
|  | P41 | FaH1P | chromosome | Fatty acid hydroxylase, putative | AII10087.1 |
|  | P96 | DavB1 | chromosome | Lysine 2-monooxygenase (EC 1.13.12.2) | AII07581.1 |
|  | P102 | DavB2 | chromosome | Lysine 2-monooxygenase (EC 1.13.12.2) | AII07957.1 |
|  | P55 | HmoA | chromosome | Antibiotic biosynthesis monooxygenase | AII03486.1 |
| **C 2 – Cyclohexanone monooxygenase s and similar enzymes** | P101 | CzcO1 | chromosome | Cyclohexanone monooxygenase (EC 1.14.13.22) | [AII07936.1](https://www.ncbi.nlm.nih.gov/protein/AII07936?report=genbank&log$=protalign&blast_rank=1&RID=FXG2TJXW014) |
|  | P53 | CzcO2 | chromosome | Cyclohexanone monooxygenase (EC 1.14.13.22) | [AII03309.1](https://www.ncbi.nlm.nih.gov/protein/AII03309?report=genbank&log$=protalign&blast_rank=1&RID=FXG2TJXW014) |
|  | P54 | CzcO3 | chromosome | Cyclohexanone monooxygenase (EC 1.14.13.22) | [AII03325.1](https://www.ncbi.nlm.nih.gov/protein/AII03325?report=genbank&log$=protalign&blast_rank=1&RID=FXG2TJXW014) |
|  | P168 | CzcO |  | 4RG3 Cyclohexanone monooxygenase - *Rhodococcus* sp. HI-31 | [BAH56677.1](https://www.ncbi.nlm.nih.gov/protein/BAH56677?report=genbank&log$=protalign&blast_rank=3&RID=FXG2TJXW014) |
|  | P73 | CzcO4 | chromosome | Cyclohexanone monooxygenase (EC 1.14.13.22) | AII05723.1 |
|  | P116 | CzcO5 | chromosome | Cyclohexanone monooxygenase (EC 1.14.13.22) | [AII08676.1](https://www.ncbi.nlm.nih.gov/protein/AII08676?report=genbank&log$=protalign&blast_rank=11&RID=FXG2TJXW014) |
|  | P72 | CzcO6 | chromosome | Cyclohexanone monooxygenase (EC 1.14.13.22) | [AII05694.1](https://www.ncbi.nlm.nih.gov/protein/AII05694?report=genbank&log$=protalign&blast_rank=1&RID=FXG2TJXW014) |
|  | P97 | CzcO7 | chromosome | Cyclohexanone monooxygenase (EC 1.14.13.22) | [AII07703.1](https://www.ncbi.nlm.nih.gov/protein/AII07703?report=genbank&log$=protalign&blast_rank=1&RID=FXG2TJXW014) |
|  | P140 | CzcO8 | pPDG2 | Cyclohexanone monooxygenase (EC 1.14.13.22) | [AII10811.1](https://www.ncbi.nlm.nih.gov/protein/AII10811?report=genbank&log$=protalign&blast_rank=1&RID=FXG2TJXW014) |
|  | P74 | CzcO9 | chromosome | Cyclohexanone monooxygenase (EC 1.14.13.22) | CP008947.1 |
|  | P52 | CzcO10 | chromosome | 4-Hydroxyacetophenone monooxygenase | [AII03268.1](https://www.ncbi.nlm.nih.gov/protein/AII03268?report=genbank&log$=protalign&blast_rank=1&RID=FXG2TJXW014) |
|  | P71 | CzcO11 | chromosome | 4-Hydroxyacetophenone monooxygenase | [AII05631.1](https://www.ncbi.nlm.nih.gov/protein/AII05631?report=genbank&log$=protalign&blast_rank=1&RID=FXG2TJXW014) |
|  | P51 | CzcO12 | chromosome | 4-Hydroxyacetophenone monooxygenase | [AII03242.1](https://www.ncbi.nlm.nih.gov/protein/AII03242?report=genbank&log$=protalign&blast_rank=1&RID=FXG2TJXW014) |
|  | P99 | CzcO13 | chromosome | 4-Hydroxyacetophenone monooxygenase | [AII07784.1](https://www.ncbi.nlm.nih.gov/protein/AII07784?report=genbank&log$=protalign&blast_rank=1&RID=FXG2TJXW014) |
|  | P64 | CzcO14 | chromosome | Cyclohexanone monooxygenase (EC 1.14.13.22) | [AII04180.1](https://www.ncbi.nlm.nih.gov/protein/AII04180?report=genbank&log$=protalign&blast_rank=1&RID=FXG2TJXW014) |
|  | P78 | CzcO15 | chromosome | 4-Hydroxyacetophenone monooxygenase | [AII05843.1](https://www.ncbi.nlm.nih.gov/protein/AII05843?report=genbank&log$=protalign&blast_rank=1&RID=FXG2TJXW014) |
|  | P125 | CzcO16 | chromosome | 4-Hydroxyacetophenone monooxygenase | [AII09069.1](https://www.ncbi.nlm.nih.gov/protein/AII09069?report=genbank&log$=protalign&blast_rank=1&RID=FXG2TJXW014) |
|  | P69 | CzcO17 | chromosome | Cyclohexanone monooxygenase (EC 1.14.13.22) | [AII05547.1](https://www.ncbi.nlm.nih.gov/protein/AII05547?report=genbank&log$=protalign&blast_rank=1&RID=FXG2TJXW014) |
|  | P128 | CzcO18 | chromosome | Cyclohexanone monooxygenase (EC 1.14.13.22) | [AII09304.1](https://www.ncbi.nlm.nih.gov/protein/AII09304?report=genbank&log$=protalign&blast_rank=1&RID=FXG2TJXW014) |
|  | P76 | CzcO19 | chromosome | Cyclohexanone monooxygenase (EC 1.14.13.22) | CP008947.1 |
|  | P66 | CzcO20 | chromosome | 4-Hydroxyacetophenone monooxygenase | [AII05035.1](https://www.ncbi.nlm.nih.gov/protein/AII05035?report=genbank&log$=protalign&blast_rank=1&RID=FXG2TJXW014) |
|  | P87 | CzcO21 | chromosome | Cyclohexanone monooxygenase (EC 1.14.13.22) | [AII06778.1](https://www.ncbi.nlm.nih.gov/protein/AII06778?report=genbank&log$=protalign&blast_rank=1&RID=FXG2TJXW014) |
| **C 3 – FAD-dependent monooxygenases and similar enzymes** | P88 | EthA1 | chromosome | FAD-containing monooxygenase EthA | [AII06822.1](https://www.ncbi.nlm.nih.gov/protein/AII06822?report=genbank&log$=protalign&blast_rank=1&RID=FXG2TJXW014) |
|  | P108 | EthA2 | chromosome | FAD-containing monooxygenase EthA | [AII08233.1](https://www.ncbi.nlm.nih.gov/protein/AII08233?report=genbank&log$=protalign&blast_rank=1&RID=FXG2TJXW014) |
|  | P89 | Fmo1 | chromosome | Flavin-containing monooxygenase | AII06850.1 |
|  | P139 | Fmo2 | chromosome | Flavin-containing monooxygenase | AII10303.1 |
|  | P84 | Fmo3 | chromosome | Monooxygenase, putative | AII06597.1 |
|  | P100 | Fmo4 | chromosome | FAD-dependent oxidoreductase | AII07843.1 |
|  | P98 | Fmo5 | chromosome | Monooxygenase | AII07779.1 |
|  | P148 | Fmo6 | pPDG3 | FAD-dependent oxidoreductase | AII11264.1 |
|  | P181 | Fmos |  | 4USR Flavin-containing monooxygenase - *Pseudomonas stutzeri* NF13 | EME00352.1 |
|  | P104 | Dma | chromosome | FAD-dependent oxidoreductase | AII08101.1 |
| **NC 1** | P4 | DavB1 | chromosome | Lysine N6-hydroxylase (EC:1.14.13.59) | AII04026.1 |
|  | P82 | DavB2 | chromosome | L-lysine 6-monooxygenase | AII06354.1 |
|  | P62 | Fmo7 | chromosome | Monooxygenase | AII04027.1 |
|  | P50 | Fmo8 | chromosome | Monooxygenase, FAD-binding | AII03165.1 |
|  | P186 | HpaC |  | 2ECR 4-Hydroxyphenylacetate 3-monooxygenase *Thermus thermophilus* | Q5SJP7.1 |
| **C 4 – Methane monooxygenases and similar enzymes** | P155 | TmoA |  | Toluene monooxygenase hydroxylase - *Pseudomonas mendocina* KR1 | AAS66660.1 |
|  | P158 | TbuA1 |  | Toluene-3-monooxygenase oxygenase -  *Ralstonia pickettii* PKO1 | AAB09618.1 |
|  | P161 | PH |  | Phenol hydroxylase four component -  *Ralstonia eutropha* JMP134 | AAC77380.1 |
|  | P151 | TouA |  | Toluene *o*-xylene monooxygenase -  *Pseudomonas* sp. OX1 | CAA06654.1 |
|  | P156 | BmoA |  | Benzene monooxygenase -  *Pseudomonas aeruginosa* JI104 | BAA11761.1 |
|  | P157 | TbhA |  | Toluene-3-monooxygenase oxygenase – *Burkholderia cepacia* AA1 | AAB58740.1 |
|  | P154 | TbmD |  | α subunit-terminal oxygenase component - *Pseudomonas* sp. JS150 | AAA88459.1 |
|  | P152 | PhN |  | Phenol hydroxylase -  *Pseudomonas* sp. OX1 | AAO47358.1 |
|  | P159 | DmpN |  | Phenol Hydroxylase -  *Pseudomonas putida* CF600 | BAP28469.1 |
|  | P59 | PrmA | chromosome | Methane monooxygenase component A *alpha* chain (EC 1.14.13.25) | AII03499.1 |
|  | P160 | Mmox |  | Methane monooxygenase, A subunit - *Methylococcus capsulatus* | AAU92736.1 |
|  | P187 | MmoH |  | 1MHY Methane monooxygenase hydroxylase *Methylosinus trichosporium* | ATQ70364.1 |
| **NC 2** | P70 | SsuD1 | chromosome | Monooxygenase-luciferase | AII05616.1 |
|  | P106 | LuxA1 | chromosome | Alkanal monooxygenase *alpha* chain  (EC 1.14.14.3) | AII08184.1 |
|  | P105 | LuxA2 | chromosome | Alkanal monooxygenase *alpha* chain  (EC 1.14.14.3) | AII08179.1 |
| **NC 3** | P109 | - | chromosome | Putative monooxygenase | [AII08275.1](https://www.ncbi.nlm.nih.gov/protein/AII08275?report=genbank&log$=protalign&blast_rank=1&RID=FXMWECGB014) |
| **C 5 – Salicylate hydroxylases and similar enzymes** | P178 | Thi4 |  | 4Y4M Thiazole synthase *Methanocaldococcus jannaschii* | AAB98592.1 |
|  | P9 | Sal1 | chromosome | Salicylate hydroxylase (EC 1.14.13.1) | AII05373.1 |
|  | P94 | Fmo9 | chromosome | Aromatic ring hydroxylase | AII07390.1 |
|  | P174 | Sal |  | 5EVY Salicylate hydroxylase -  *Pseudomonas putida* | BAA61829.1 |
|  | P25 | Sal2 | chromosome | Salicylate hydroxylase (EC 1.14.13.1) | AII07743.1 |
|  | P20 | Sal3 | chromosome | Salicylate hydroxylase (EC 1.14.13.1) | AII07112.1 |
|  | P173 | NicC |  | 5EOW 6-Hydroxynicotinic Acid 3-monooxygenase -  *Pseudomonas putida* KT2440 | AAN69538.1 |
|  | P39 | Hba1 | chromosome | Putative *n*-hydroxybenzoate hydroxylase | AII09308.1 |
|  | P153 | Hba |  | Salicylate monooxygenase –  *Rhodococcus jostii* RHA1 | ABG93680.1 |
| **NC 4** | P31 | HpaB4 | chromosome | 4-Hydroxyphenylacetate 3-monooxygenase  (EC 1.14.13.2) | AII08627.1 |
|  | P180 | HbpA |  | 4CY6 2-Hydroxybiphenyl 3-monooxygenase -  *Pseudomonas nitroreducens* | AAB57640.1 |
|  | P1 | - | chromosome | Aromatic ring hydroxylase | AII03285.1 |
|  | P147 | Fmo10 | pPDG2 | Monooxygenase, FAD-binding | CP008949.1 |
|  | P91 | Fmo11 | chromosome | 2-polyprenyl-6-methoxyphenol hydroxylase | AII07178.1 |
|  | P75 | Fmo12 | chromosome | FAD-dependent monooxygenase | CP008947.1 |
|  | P81 | Fmo13 | chromosome | Monooxygenase | AII06159.1 |
|  | P38 | MhaA1 | chromosome | 2-Polyprenyl-6-methoxyphenol hydroxylase and related FAD-dependent oxidoreductases | AII09237.1 |
|  | P61 | Fmo14 | chromosome | FAD-dependent oxidoreductases | AII03943.1 |
|  | P14 | MhaA2 | chromosome | Pentachlorophenol monooxygenase | AII06154.1 |
|  | P103 | MhpA1 | chromosome | 3-(3-hydroxyphenyl)propinate hydroxylase | AII08000.1 |
|  | P119 | Fmo15 | chromosome | Aromatic ring hydroxylase | AII08743.1 |
|  | P3 | MhpA2 | chromosome | 3-(3-Hydroxy-phenyl)propionate hydroxylase  (EC 1.14.13.-) | CP008947.1 |
|  | P6 | MhpA3 | chromosome | 3-(3-Hydroxy-phenyl)propionate hydroxylase  (EC 1.14.13.-) | CP008947.1 |
|  | P129 | MhpA4 | chromosome | 3-(3-hydroxyphenyl)propionate hydroxylase | AII09317.1 |
|  | P40 | HpnW | chromosome | Probable aromatic ring hydroxylase | AII09332.1 |
|  | P23 | MhaA3 | chromosome | Pentachlorophenol monooxygenase | AII07569.1 |
| **NC 5** | P49 | - | chromosome | Putative dioxygenasehydroxylase component | AII10950.1 |
| **C 6 – Putative cytochrome P450 hydroxylases** | P24 | CypX1 | chromosome | Putative cytochrome P450 hydroxylase | AII07617.1 |
|  | P35 | CypX2 | chromosome | Putative cytochrome P450 hydroxylase | AII08744.1 |
|  | P28 | CypX3 | chromosome | Putative cytochrome P450 hydroxylase | AII08421.1 |
|  | P29 | CypX4 | chromosome | Putative cytochrome P450 hydroxylase | AII08505.1 |
|  | P21 | CypX5 | chromosome | Putative cytochrome P450 hydroxylase | AII07296.1 |
|  | P43 | CypX6 | chromosome | Putative cytochrome P450 hydroxylase | AII10559.1 |
|  | P44 | CypX7 | chromosome | Putative cytochrome P450 hydroxylase | AII10827.1 |
|  | P16 | CypX8 | chromosome | Putative cytochrome P450 hydroxylase | AII06401.1 |
|  | P37 | CypX9 | chromosome | Putative cytochrome P450 hydroxylase | AII08831.1 |
|  | P8 | CypX10 | chromosome | Putative cytochrome P450 hydroxylase | AII05330.1 |
|  | P17 | CypX11 | chromosome | Putative cytochrome P450 hydroxylase | AII06482.1 |
|  | P15 | CypX12 | chromosome | Putative cytochrome P450 hydroxylase | AII06399.1 |
|  | P30 | CypX13 | chromosome | Putative cytochrome P450 hydroxylase | AII08534.1 |
|  | P47 | CypX14 | chromosome | Putative cytochrome P450 hydroxylase | AII10868.1 |
|  | P2 | CypX15 | chromosome | Putative cytochrome P450 hydroxylase | AII03447.1 |
|  | P45 | CypX16 | chromosome | Putative cytochrome P450 hydroxylase | AII10849.1 |
|  | P46 | CypX17 | chromosome | Putative cytochrome P450 hydroxylase | AII10849.1 |
|  | P42 | CypX18 | chromosome | Putative cytochrome P450 hydroxylase | AII10517.1 |
|  | P5 | CypX19 | chromosome | Putative cytochrome P450 hydroxylase | AII04108.1 |
|  | P33 | CypX20 | chromosome | Putative cytochrome P450 hydroxylase | AII08657.1 |
|  | P26 | CypX21 | chromosome | Putative cytochrome P450 hydroxylase | AII07750.1 |
|  | P27 | CypX22 | chromosome | Putative cytochrome P450 hydroxylase | AII08206.1 |
|  | P10 | CypX23 | chromosome | Putative cytochrome P450 hydroxylase | CP008947.1 |
| **C 7 – Nitrilotriacetate monooxygenases and similar enzymes** | P124 | NmoA1 | chromosome | Nitrilotriacetate monooxygenase component A (EC 1.14.13.-) | AII08959.1 |
|  | P132 | NmoA2 | chromosome | Nitrilotriacetate monooxygenase component A (EC 1.14.13.-) | AII09422.1 |
|  | P170 | Nmo |  | 3SDO Nitrilotriacetatemonooxygenase - *Burkholderia pseudomallei* | ABA52898.1 |
|  | P138 | NmoA3 | chromosome | Nitrilotriacetatemonooxygenase component A (EC 1.14.13.-) | AII09502.1 |
|  | P171 | LadA |  | 3B9N Long-chain alkane monooxygenase -*Geobacillus thermodenitrificans* | ABO68832.1 |
|  | P60 | NmoA4 | chromosome | Nitrilotriacetate monooxygenase component A | AII03608.1 |
|  | P112 | NmoA5 | chromosome | Nitrilotriacetate monooxygenase component A | AII08556.1 |
|  | P172 | EmoA |  | 5DQP EDTA monooxygenase -  *Chelativorans* sp. BNC1 | AAG09252.1 |
|  | P134 | NmoA6 | chromosome | Putative nitrilotriacetate monooxygenase, component A | AII09433.1 |
|  | P137 | NmoA7 | chromosome | Probable nitrilotriacetate monooxygenase, component A | AII09445.1 |
|  | P145 | NmoA8 | pPDG2 | Nitrilotriacetate monooxygenase component A (EC 1.14.13.-) | AII10963.1 |
|  | P68 | NmoA9 | chromosome | Nitrilotriacetate monooxygenase component A (EC 1.14.13.-) | AII05536.1 |
|  | P79 | NmoA10 | chromosome | Nitrilotriacetate monooxygenase component A (EC 1.14.13.-) | AII06110.1 |
|  | P80 | - | chromosome | Putative monooxygenase | AII06121.1 |
| **C 8 – Alkanesulfonate monooxygenases and similar enzymes** | P146 | SsuD2 | pPDG2 | Alkanal monooxygenase *alpha* chain  (EC 1.14.14.3) | AII10970.1 |
|  | P90 | Llm | chromosome | F420-dependent oxidoreductase | AII07075.1 |
|  | P113 | AlkB | chromosome | Alkane-1-monooxygenase (EC 1.14.15.3) | AII08632.1 |
|  | P107 | RutA | chromosome | Predicted monooxygenase RutA in novel pyrimidine catabolism pathway | AII08186.1 |
|  | P120 | SfnG1 | chromosome | Alkanesulfonate monooxygenase | AII08749.1 |
|  | P131 | SfnG2 | chromosome | Alkanesulfonate monooxygenase | AII09418.1 |
|  | P188 | Lux |  | 3RAO Luciferase-like Monooxygenase - *Bacillus cereus* ATCC 10987 | AAS39998.1 |
|  | P63 | SsuD3 | chromosome | Alkanesulfonate monooxygenase (EC 1.14.14.5) | CP008947.1 |
|  | P121 | SsuD4 | chromosome | Alkanesulfonate monooxygenase (EC 1.14.14.5) | AII08753.1 |
|  | P183 | SsuD |  | 1M41 Alkanesulfonate monooxygenase - *Escherichia coli* | NP_415455.1 |
| **C 9 – Putative monooxygenases/hydroxylases and similar enzymes** | P22 | - | chromosome | Putative hydroxylase | AII07316.1 |
|  | P11 | - | chromosome | Putative hydroxylase | AII05855.1 |
|  | P19 | - | chromosome | Putative hydroxylase | AII06974.1 |
|  | P34 | - | chromosome | Hydroxylase | AII08679.1 |
|  | P7 | - | chromosome | Hydroxylase | AII05251.1 |
|  | P12 | - | chromosome | Hydroxylase | AII05997.1 |
|  | P176 | DszC |  | 3X0X Dibenzothiophene monooxygenase - *Rhodococcus erythropolis* D-1 | BAQ25859.1 |
|  | P177 | DszC |  | 4NXL Dibenzothiophene monooxygenase - *Rhodococcus erythropolis* | AAU14822.1 |
|  | P175 | DnmZ |  | 4ZXV Acyl-CoA dehydrogenase - *Streptomyces peucetius* | ATW50550.1 |
| **C 10– Ubiquinone monooxygenases and similar enzymes** | P114 | HmoA1 | chromosome | Antibiotic biosynthesis monooxygenase | AII08639.1 |
|  | P65 | UbiB1 | chromosome | Ubiquinone biosynthesis monooxygenase | AII04651.1 |
|  | P118 | UbiB2 | chromosome | Ubiquinone biosynthesis monooxygenase | AII08725.1 |
| **NC 6** | P85 | HmoA2 | chromosome | Antibiotic biosynthesis monooxygenase | AII06649.1 |
|  | P126 | Fmo16 | chromosome | Possible flavin binding monooxygenase | AII09070.1 |
|  | P13 | EctD1 | chromosome | Hydroxylase | AII06134.1 |
|  | P18 | EctD2 | chromosome | Hydroxylase | AII06497.1 |
|  | P163 | Mmo |  | Oxidoreductase FAD-binding domain protein -*Pseudomonas* sp. Chol1 | EKM96312.1 |
|  | P36 | DdhD1 | chromosome | 2-Polyprenylphenol hydroxylase and related flavodoxin oxidoreductases / CDP-6-deoxy-delta-3,4-glucoseen reductase-like | AII08803.1 |
|  | P48 | DdhD2 | chromosome | 2-Polyprenylphenol hydroxylase and related flavodoxin oxidoreductases / CDP-6-deoxy-delta-3,4-glucoseen reductase-like | CP008949.1 |
|  | P162 | TomA1 |  | Toluene/phenol hydroxylase - *Burkholderia cepacia* G4 | AAK07409.1 |
|  | P77 | AmoA | chromosome | Putative ammonia monooxygenase | AII05831.1 |
|  | P136 | NmoA11 | chromosome | Nitrilotriacetatemonooxygenase | AII09443.1 |
|  | P135 | NmoA12 | chromosome | Probable nitrilotriacetate monooxygenase, component A | AII09437.1 |
|  | P95 | - | chromosome | Putative monooxygenase | AII07476.1 |
