# Supplementary material for: Genome-based analysis for the identification of genes involved in o-xylene degradation in Rhodococcus opacus R7
Source: BMC Genomics. 2018 Aug 6;19:587. doi: 10.1186/s12864-018-4965-6 (PMC6080516; doi:10.1186/s12864-018-4965-6)
Supplement: Supplementary file 3 — Table S3. List of utilized oligos. (DOCX 15 kb) [file 12864_2018_4965_MOESM3_ESM.docx]

**Table S3. List of oligonucleotides used for RT-PCR and qRT-PCR**

| **Oligonucleotide name** | **Sequence (5’ – 3’)** | **Melting Temperature (T_m_)** |
| --- | --- | --- |
| **27f** | AGAGTTTGATCCTGGCTCAG | 55°C |
| **1495r** | CTACGGCTACCTTGTTACGA | 55°C |
| **akbA1-GTG-for** | GTGAATCCGCAGGACGGGTGG | 51°C |
| **akbA2-TGA-rev** | TCAGAGGAAGAGGTTGAGATT | 51°C |
| **akbB-ATGfor** | ATGGGTTGGCTGGAAGACAATG | 54°C |
| **akbB-TAGrev** | CTAAAGGTCATTTCCGCCAGC | 54°C |
| **akbC-ATGfor** | ATGACAAAAGTGACCGAGCTC | 53°C |
| **akbC-TAGrev** | CTACTTGAGGGGGATATCCAAG | 53°C |
| **prmAf-TTG** | TTGAGTAGGCAAAGCCTGACA | 57°C |
| **prmAr-TGA** | TCAGGCCGGAACTGTGCCGCC | 57°C |
| **PheA1-ATG-for** | ATGACCACCACCGAATCCGCC | 60°C |
| **PheA1-CTA-rev** | CTAGCTGCGGCCGAAGTAGGA | 60°C |
| **Oligonucleotide name** | **Sequence (5’ – 3’)** | **Melting Temperature (T_m_)** |
| **RT-16S-R7f** | TCGTGAGATGTTGGGTTAAG | 55°C |
| **RT-16S-R7r** | CCTCTGTACCGGCCATTGTAG | 62°C |
| **RT-AkbA1-f** | ATATGATCTTGGACAATGAGG | 54°C |
| **RT-AkbA1-r** | ATTCTCCATATCAATCTCGGG | 56°C |
| **RT-PrmA-f** | AACATCTACCTGACCGTGGT | 57°C |
| **RT-PrmA-r** | GCCATCAGCAGGATCGAATA | 54°C |
| **RT-PheA1-f** | TGAAACTCGACTTCATCGCC | 57°C |
| **RT-PheA1-r** | TATTCGAGCTTCGGGATGAC | 57°C |
