# Supplementary material for: Genome-based analysis for the identification of genes involved in o-xylene degradation in Rhodococcus opacus R7
Source: BMC Genomics. 2018 Aug 6;19:587. doi: 10.1186/s12864-018-4965-6 (PMC6080516; doi:10.1186/s12864-018-4965-6)
Supplement: Supplementary file 4 — Table S4. List of utilized oligos for transposon identification. (DOCX 13 kb) [file 12864_2018_4965_MOESM4_ESM.docx]

**Table S4. List of oligonucleotides used for Two-Step gene walking PCR amplification**

| **Oligonucleotide name** | **Sequence (5’ – 3’)** | **Melting Temperature (T_m_)** |
| --- | --- | --- |
| Walking_thio_1 | GGAAAAGGACTGCTGTCGCTGCC | 63°C |
| i-pTipThio-70-rev | CAAGGGGAAGTCGTCGCTCTCTGG | 66°C |
| pTNR884-for | TTGGTAGCTCTTGATCCGGCAAAC | 72°C |
| Walking_3-for | AACAACTGGCCGCCACC | 68°C |
